# A Population-Based (Super-Child) Approach for Predicting Vitamin A Total Body Stores and Retinol Kinetics in Children Is Validated by the Application of Model-Based Compartmental Analysis to Theoretical Data

**DOI:** 10.1093/cdn/nzy071

**Published:** 2018-11-24

**Authors:** Jennifer Lynn Ford, Joanne Balmer Green, Michael H Green

**Affiliations:** Department of Nutritional Sciences, College of Health and Human Development, The Pennsylvania State University, University Park, PA

**Keywords:** high vitamin A intake, humans, nutrition assessment, retinol isotope dilution, theoretical children, vitamin A kinetics, vitamin A status, WinSAAM

## Abstract

**Background:**

Public health nutritionists need accurate and feasible methods to assess vitamin A status and to evaluate efficacy of interventions, especially in children. The application of population-based designs to tracer kinetic data is an effective approach that reduces sample burden for each child.

**Objectives:**

Objectives of the study were to use theoretical data to validate a population-based (super-child) approach for estimating group mean vitamin A total body stores (TBS) and retinol kinetics in children and to use population-based data to improve individual TBS predictions using retinol isotope dilution (RID).

**Methods:**

We generated plasma retinol kinetic data from 6 h to 56 d for 50 theoretical children with high vitamin A intakes, assigning values within physiologically reasonable ranges for state variables and kinetic parameters (“known values”). Mean data sets for all subjects at extensive (*n* = 36) and reduced (*n* = 11) sampling times, plus 5 data sets for reduced numbers (5/time, except all at 4 d) and times, were analyzed using Simulation, Analysis and Modeling software. Results were compared with known values; population RID coefficients were used to calculate TBS for individuals.

**Results:**

For extensive and reduced data sets including all subjects, population TBS predictions were within 1% of the known value. For 5 data sets reflecting numbers and times being used in ongoing super-child studies, predictions were within 1–17% of the known group value. Using RID equation coefficients from population modeling, TBS predictions at 4 d were within 25% of the known value for 66–80% of subjects and reflected the range of assigned values; when ranked, predicted and assigned values were significantly correlated (*R_s_* = 0.93, *P *< 0.0001). Results indicate that 7 d may be better than 4 d for applying RID in children. For all data sets, predictions for kinetic parameters reflected the range of known values.

**Conclusion:**

The population-based (super-child) approach provides a feasible experimental design for quantifying retinol kinetics, accurately estimating group mean TBS, and predicting TBS for individuals reasonably well.

## Introduction

Vitamin A deficiency, especially in children, remains a public health concern in many parts of the world, despite the fact that vitamin A supplementation has been shown to be effective in reducing child morbidity and mortality (1). Large-scale interventions continue to be implemented in at-risk populations, but some researchers worry that the availability of multiple supplementation programs may expose children to excessive amounts of vitamin A (2). Thus, it is critical that accurate and feasible methods are available for assessing vitamin A status in this age group, at both the population and individual levels. At present, the retinol isotope dilution (RID) technique is considered the best-available method to evaluate vitamin A status in the field (3); it is also one of the few methods assumed to be valid for assessing status over a very wide range of vitamin A stores (4). However, the accuracy of RID equations for predicting vitamin A total body stores (TBS) relies on assumptions about absorption and retention of the tracer dose and about isotope mixing; these are included as coefficients in the prediction equations (5). In addition, this method is generally considered valid for groups but not for individual subjects (4).

Although not yet widely used for this purpose, model-based compartmental analysis (6) offers another strategy for estimating vitamin A TBS. The method has already been fruitfully applied by Green and colleagues (6–14) to describe and quantify whole-body vitamin A kinetics and compartment masses in rats (6–9), monkeys (10), and humans (11–14). One drawback to this approach is that it requires repeated blood sampling over time and thus is not ideal for use in children. A feasible alternative strategy is population-based modeling. This approach, which involves limited sampling of each subject followed by analysis of a composite data set, is frequently used in pharmacokinetics (15, 16). We previously used a population-based (super-rat/super-child) approach to develop whole-body models of vitamin A metabolism in rats (7, 17) and, in collaboration with Haskell et al. (18), to estimate vitamin A fractional catabolic rate and equilibration time in Peruvian children. More recently, we applied a super-child approach in collaboration with Lopez-Teros et al. (14) to estimate vitamin A TBS and to describe retinol kinetics in Mexican children. In addition, the approach is being used in ongoing experiments funded by the Bill & Melinda Gates Foundation and the International Atomic Energy Agency to evaluate vitamin A status in children exposed to multiple sources of the nutrient (e.g., high-dose supplements, fortified foods, and micronutrient powders). In studies in Bangladesh, Guatemala, Morocco, Nigeria, Philippines, and Zambia, children (*n* = 50) ingest a dose of stable isotope–labeled retinyl acetate; a blood sample is collected from all children 4 d later, and a second sample is obtained from each child at 1 of 10 additional times between 6 h and 28 d. Model-based compartmental analysis [specifically, WinSAAM, the Windows version of the Simulation, Analysis and Modeling software (19, 20)] will then be applied to each study's composite data set for plasma retinol isotope response versus time to estimate vitamin A TBS and retinol kinetics for the population. Modeling results will also be used to generate population-specific estimates for the coefficients used in the 4-d RID equation (21) and vitamin A TBS will be calculated for individual children.

In the current work, we generated and analyzed a theoretical data set using compartmental analysis to test the hypothesis that the super-child approach provides accurate predictions of population vitamin A TBS and retinol kinetics as well as population-specific values for RID equation coefficients for estimating TBS for individuals.

## Methods

### Overview of approach

**FIGURE 1:**
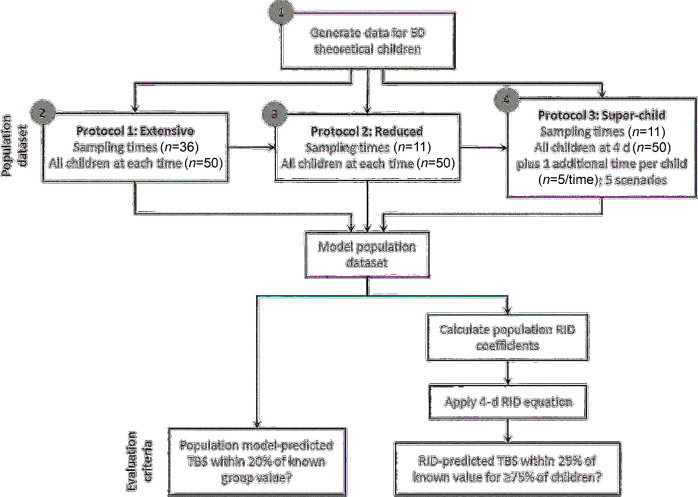
Flow chart of the theoretical analysis used to evaluate the super-child approach. A database for 50 theoretical children with known values for vitamin A TBS and other variables was used to generate population (mean) data sets for 3 sampling protocols. For each data set, model-based compartmental analysis was applied to estimate vitamin A TBS and retinol kinetics for the group. Population estimates for RID equation coefficients were calculated from the model and used to predict vitamin A TBS at 4 d for individual subjects. Predictions of TBS were compared with known values using the specified evaluation criteria. Circled numbers correspond to the following objectives: *1*) use model-based compartmental analysis to generate a database of known values for theoretical subjects that was needed to test the population-based approach, *2*) establish proof-of-concept that population-based modeling of a detailed data set provides accurate estimates for the variables of interest, *3*) validate the approach using reduced sampling times, and *4*) validate the super-child design being implemented in ongoing super-child studies. RID, retinol isotope dilution; TBS, total body stores.

Our objectives were to evaluate a population-based (super-child) approach both for estimating group mean vitamin A TBS and retinol kinetics and for predicting individual values for TBS by analyzing a theoretical database and comparing those results with known values. As an overview and as described in the next section and in **Supplemental Methods**, we first used model-based compartmental analysis to generate a database that included 50 theoretical children with a wide range of known values for whole-body retinol kinetic parameters and specified state variables such as vitamin A TBS, plasma retinol pool size, and dietary vitamin A intake. We use the term “known values” as it is understood in pharmacokinetics (15) to describe information that is assigned or calculated a priori for many theoretical individuals and then used for validation of population data sets (see references 22 and 23 for applications in the vitamin A field). Next, we used the known values for model parameters as inputs into a compartmental model for vitamin A metabolism and used WinSAAM to simulate plasma retinol kinetic response data for each individual. Then, as shown in Figure 1, population (mean) data sets were generated for 3 sampling protocols: protocol 1 included all 50 subjects and an extensive sampling schedule (36 samples over 56 d) to establish proof-of-concept for the population-based approach, protocol 2 included all subjects but a reduced sampling schedule (11 samples over 28 d) to extend the verification when using limited sampling, and protocol 3 mimicked the super-child design being applied in ongoing field studies, as described in the Introduction, using the reduced number of sampling times as well as a reduced number of blood samples from each subject (2 samples/child). We used model-based compartmental analysis to analyze each composite data set and obtained population-based predictions of vitamin A TBS and retinol kinetics as well as population estimates for RID equation coefficients that were used to calculate vitamin A TBS for each individual. To evaluate the accuracy of results for each protocol, population-based predictions of TBS and retinol kinetics were compared with known values for the group and individual TBS predictions were compared with the known value for each child.

### Generation of theoretical data for individuals

**FIGURE 2:**
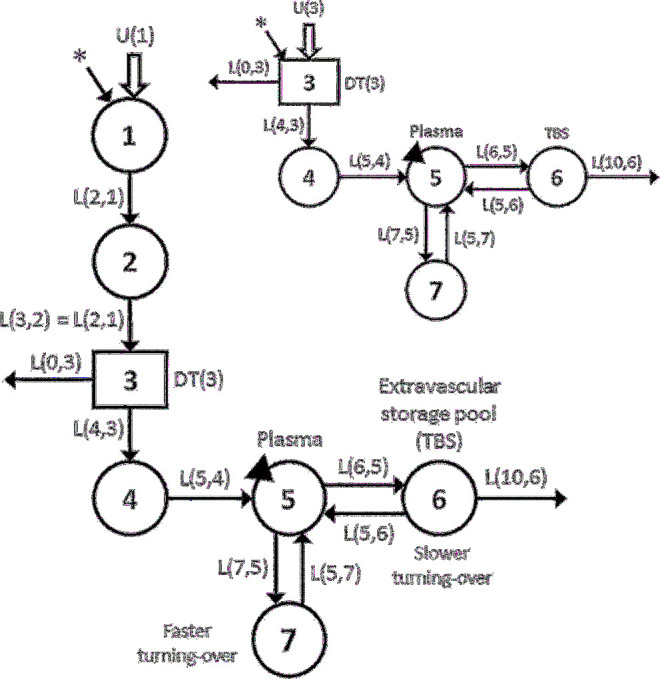
Working compartmental model for retinol kinetics in children. Circles represent compartments; the rectangle is a delay component and DT(3) is the delay time retinol spent in component 3; and interconnectivities between compartments (arrows) are fractional transfer coefficients [L(I,J)s, or the fraction of retinol in compartment J transferred to compartment I each day]. For example, L(6,5) is the fraction of retinol in compartment 5 transferred to compartment 6 each day and L(5,6) is the fraction in compartment 6 transferred to compartment 5 each day. Compartment 1 is the site of input of orally ingested tracer (*) and dietary vitamin A [U(1)]; fractional absorption efficiency was fixed at 0.80. Components 1–4 represent the processing of preformed dietary vitamin A, chylomicron production and metabolism, hepatic uptake of chylomicron remnant retinyl esters, and processing of retinol (compartment 4); delay component 3 is the site of loss of unabsorbed tracer. Retinol is secreted from the liver bound to retinol-binding protein into plasma compartment 5, the site of sampling (triangles). Retinol in the plasma pool can exchange with vitamin A in 2 extravascular compartments: a faster turning-over pool (compartment 7) and a slower turning-over storage pool (compartment 6), which is also the site of irreversible loss from the system. The inset shows the simplified model used to analyze the reduced data sets (protocols 2 and 3). In the simplified model, delay component 3 is the site of input for tracer (*) and dietary vitamin A [U(3)]. TBS, total body stores.

First, we articulated the 7-compartment model shown in Figure 2 on the basis of models previously developed for adults (12, 13) and children (14). Compartments 1–4 (including delay component 3) represent the processing of dietary vitamin A until it is taken up by the hepatocytes (compartment 4) and secreted into plasma compartment 5 as retinol bound to retinol-binding protein. Retinol in plasma can exchange with vitamin A in 2 extravascular compartments: a slower turning-over pool (compartment 6; vitamin A TBS), which is also the site of irreversible loss from the system, and a faster turning–over pool (compartment 7). Physiologically, compartment 7 likely includes vitamin A in tissues that do not function as long-term storage sites and which turn over vitamin A faster. A faster turning–over extravascular pool has been used to better fit plasma retinol kinetic data in some previous studies (11, 14) but not in others (12, 13). In light of these previous findings, we generated data for some subjects (*n* = 23) using a model with one extravascular storage pool (compartment 6), whereas for others (*n* = 27) we used a model with 2 extravascular compartments. Then, for each theoretical subject, we populated the model with the known values for the model parameters, including time spent in the delay component [DT(3)] and fractional transfer coefficients [L(I,J)s, or the fractional transfer of retinol in compartment J to compartment I each day]. To establish physiologically reasonable known values for kinetic parameters, we reviewed published data, including observations that retinol turnover and recycling are faster in children and young animals than in adults (9, 12–14). We also established physiologically reasonable ranges for TBS (90–2000 µmol), dietary vitamin A intake [600–5000 µg retinol activity equivalents (RAE)/d], and other variables and then assigned values to each child. Note that the range we used for vitamin A intake is higher than the Tolerable Upper Intake Level (600 µg RAEs/d) for young children because we wanted to mimic the ongoing trials (see Introduction), which are targeting populations with anticipated higher intakes (2). Because we hypothesized that subjects had adapted to these chronic high intakes, the model used for this work assumed a balanced (steady) state (see **Supplemental Results** for details). Additional details on assignment and calculation of kinetic parameters and other variables are provided in Supplemental Methods.

Then, we used the assigned values for model parameters in WinSAAM version 3.3.0 [http://www.winsaam.org (19, 20, 24)] to simulate the fraction of the administered dose in plasma (FD_p_) from 6 h to 56 d for labeled retinyl acetate–derived retinol for each theoretical child. In empirical studies, such data would be obtained by collection and analysis of serial blood samples, whereas for the current theoretical analysis, “observed data” were simulated using the kinetic parameters specified as known values for each subject. Finally, we fixed values for plasma retinol pool size [M(5); Figure 2] and calculated a steady state solution in WinSAAM to obtain the other compartment masses and transfer rates. We confirmed that, for each subject, the model accurately predicted the vitamin A masses assigned for compartments 6 [M(6); TBS] and 7 [M(7)].

### Generation of population data sets

To evaluate the use of population-based modeling for predicting retinol kinetic parameters and key state variables such as TBS, we designed 3 protocols and then generated population data sets for each (Figure 1). Protocol 1 included data for all theoretical children (*n* = 50) at the times (*n* = 36) specified in the preceding section and in Supplemental Methods, providing an extensive sampling data set. Protocol 2 included data for all 50 children at the 11 sampling times that are being used in ongoing studies (6, 9, and 12 h and 1, 2, 4, 7, 11, 16, 22, and 28 d). These times were initially determined by sensitivity analysis (25), with adjustments made for the current field studies. For protocol 3, 5 population data sets were generated using the reduced number of sampling times (*n* = 11) with limited subject numbers as in the ongoing super-child studies; this protocol included FD_p_ data for all 50 children on day 4 and for 5 different subjects at each of the remaining 10 times. Randomization of children to their second (non–4 d) time in protocol 3 was repeated 5 times to generate 5 super-child data sets for analysis; this strategy allowed us to determine whether collecting and analyzing 2 blood samples/child would adequately capture the central tendency for the group and whether arbitrary assignment of the second sampling time would influence the results. Note that, although the theoretical data used in protocols 1 and 2 are error-free, both the randomization of subjects into the 5 super-child data sets in protocol 3 and the range of kinetics used to generate individual subject tracer response data introduce “error” into these latter data sets, making the data more comparable to what has been observed in the field.

Then, for each data set in the 3 protocols, we used Equation *1* to calculate geometric mean FD_p_:

(1) \begin{eqnarray*} {\rm{Geometric}}\, {{\rm mean\,FD}_{{\rm p}(t)}} = {\rm{antilog}}\left\{ {\left[ {\sum\nolimits_{(i = 1)}^n {{\rm{log}}\left( {{\rm{F}}{{\rm{D}}_{p\left( i \right)}}} \right)} } \right]{\rm{ \div }}n} \right\} \end{eqnarray*}

where FD_p_ is “observed” (i.e., model-generated) data for the fraction of dose in plasma, *i* is the *i*th subject, and *n* is the sample size used at time *t*. Geometric means have been used in previous vitamin A modeling studies (14, 17).

### Estimation of TBS and whole-body retinol kinetics using compartmental modeling

For each of the data sets described in the section above, population data for geometric versus time were plotted and fit to a compartmental model using WinSAAM. Specifically, data from the extensive sampling protocol (protocol 1) were fit to the full physiologic model (Figure 2), whereas the reduced data sets (protocols 2 and 3) were fit to the simplified model shown in the inset to Figure 2. Note that, in previous human studies (12, 13), the kinetic parameters defined by the initial portion of the full model (compartments 1 and 2) were generally not well identified and, other than the fraction of the dose absorbed, these parameters do not influence model predictions of TBS or postabsorptive retinol kinetics. Thus, for analyzing the reduced data sets in which early data (<1 d) are limited, we used the simplified model. In the simplified model, inputs of dietary preformed vitamin A [U(3)] and the labeled retinyl acetate dose were directly into delay component 3. In both models, retinol absorption efficiency was fixed at 80%, the value used by Lopez-Teros et al. (14) and also the midpoint of the range used in generating our theoretical data. To facilitate comparisons of the full with simplified models for processes that occur until retinol enters plasma bound to retinol-binding protein, we calculated MST_RBP_, the mean sojourn time for the orally administered labeled retinol to appear in plasma compartment 5 (i.e., the time required for dietary vitamin A to be transported through the gastrointestinal tract, absorbed and incorporated into chylomicrons, cleared by the liver, and secreted into plasma as retinol bound to retinol-binding protein). In addition, because data for some of the theoretical subjects had been generated using a model with one extravascular compartment (compared with 2 for others), we determined whether the geometric mean data for each population data set were best fit using the model with 1 or 2 extravascular compartments; results of this analysis are provided in Supplemental Results. To determine which model was best, we specified that an increase in model complexity was justified only when it significantly (*P* < 0.05) improved the weighted sums of squares using an F-statistic (26) and/or provided population model predictions of TBS that were closer to the known value for the group (see evaluation criterion described at the end of this section).

For each population data set, once a satisfactory fit was obtained between the geometric mean FD_p_ data and the model predictions, weighted nonlinear regression analysis was used to estimate final model parameters and their statistical uncertainty (24). For protocols 1 and 2, a fractional SD of 0.01 was used as a weighting factor at all times. For the 5 super-child data sets (protocol 3) that included only 5 children at each time except at 4 d, data were modeled using a fractional SD of 0.05 at all times except at 4 d, when we used 0.01. After final parameters had been obtained, we calculated the geometric mean plasma retinol pool size [M(5)] and used it in a steady state solution in WinSAAM to estimate the other compartment pool sizes, including vitamin A TBS [M(6)] and transfer rates.

Finally, for each protocol, we compared the population model-predicted TBS with the geometric mean of the assigned TBS values (i.e., known group TBS). As an evaluation criterion, we specified that a model-predicted TBS within 20% of the known value for the group would be considered accurate. In addition, for each protocol, we compared other population kinetic parameters predicted by the model with the known values.

### Prediction of vitamin A TBS by RID

RID equations have been developed to estimate TBS (for reviews, see references 3, 5, and 27). After oral administration of isotope-labeled vitamin A, plasma retinol specific activity (SA_p_) is determined after the isotope has mixed with endogenous vitamin A in the body's exchangeable pools. In addition to SA_p_, RID equations include coefficients that account for absorption and retention of the dose and mixing of tracer with the body pool. Here we used the RID equation presented by Green et al. (21), shown below as Equation *2*, to predict TBS for individual subjects:

(2) \begin{eqnarray*} {\rm{TBS}}\left( {\rm{\mu}} {\rm{mol}} \right) = Fa \times S \times 1/{\rm{S}}{{\rm{A}}_{\rm{p}}} \end{eqnarray*}

where *Fa* is the fraction of dose of labeled vitamin A absorbed and found in the body's storage pool at time *t*, *S* is the ratio of retinol specific activity in plasma to that in the storage pool at time *t*, and SA_p_ was calculated as FD_p_ ÷ plasma retinol pool size (µmol) at time *t*. In empirical studies, one would calculate SA_p_ as [tracer ÷ (tracer + tracee)] ÷ dose (µmol) in plasma at time *t*. We used Equation *2* as follows to predict TBS in individual children at the time (4 d) that is currently being used in the ongoing field studies. First, for each protocol, we simulated values for the time-variant coefficients *Fa* and *S* at 4 d using the population model (for details on the calculation of equation coefficients by the model, see references 14 and 21 and Supplemental Methods). Population estimates for the composite coefficient *Fa* × *S* were then used in Equation *2*, along with each theoretical child's SA_p_ at 4 d, to predict TBS. Estimates were compared with the assigned value of TBS for each subject. As an evaluation criterion, we considered TBS predictions within 25% of the assigned value for ≥75% of subjects to be adequate. This assessment was performed for each population data set used in the 3 protocols.

### Statistical analyses

Data are presented as geometric means (ranges) unless otherwise specified. Data were managed with the use of Microsoft Excel. Linear least-squares regression analysis was performed with the use of GraphPad Prism 7.0 for Windows; the coefficient of determination (*R*^2^) and Spearman rank-order correlation coefficients (*R_s_*) were determined as part of this analysis. *P* < 0.05 was considered significant.

## Results

### Known values for vitamin A TBS and retinol kinetics

Known values for vitamin A TBS and other state variables, and for retinol kinetic parameters, are presented in **Supplemental Table 1** for the 50 theoretical children; selected geometric means and ranges are shown in Table 1. The mean of the assigned values for TBS [M(6)] was 538 µmol (range: 92–1904 µmol), translating to liver vitamin A concentrations ranging from 0.28 to 2.5 µmol/g (79–722 µg/g), based on the assumptions that liver is 3% of body weight for this age group and that 80% of total body vitamin A is stored in the liver (28). Known values for dietary vitamin A intake ranged from 658 to 4862 µg RAEs/d (mean: 1164 µg RAEs/d).

**TABLE 1:** Known values for retinol kinetic parameters and state variables as well as model predictions for protocol 3^1^

		Model-predicted value				
	Known value, geometric mean (range)	Scenario 1	Scenario 2	Scenario 3	Scenario 4	Scenario 5
Compartment masses, µmol						
M(5)	1.02 (0.345–2.62)	1.02	1.02	1.02	1.02	1.02
M(6)	538 (92–1904)	445	534	489	573	505
M(7)	19.3 (5.24–38.6)	6.93	19.5	10.3	13.6	10.2
Dietary input, µmol/d	4.07 (2.34–16.8)	8.63	5.09	8.67	4.08	6.85
Disposal rate, µmol/d	3.21 (1.82–12.3)	6.90	4.07	6.93	3.26	5.48
Model parameters						
MST_RBP_, d	0.522 (0.364–1.08)	0.656	0.502	0.617	0.710	0.622
L(6,5), d^−1^	30.1 (20.1–39.9)	35.2	43.5	31.7	30.0	31.5
L(5,6), d^−1^	0.0502 (0.0156–0.122)	0.0650	0.0751	0.0518	0.0476	0.0527
L(10,6), d^−1^	0.00597 (0.00201–0.210)	0.0155	0.00762	0.0142	0.00570	0.0109
L(7,5), d^−1^	31.9 (5.40–60.9)	6.01	14.4	9.31	5.51	5.55
L(5,7), d^−1^	1.67 (0.401–3.75)	0.882	0.750	0.923	0.410	0.554

1 Values are geometric means (ranges) for the known values for 50 theoretical children as well as the model-predicted values for the 5 super-child data sets (protocol 3; scenarios 1–5) that included data for all subjects on day 4 and for 5 randomly selected subjects at each of the other 10 reduced sampling times. Note that, when fitting scenario 4 data, the geometric mean dietary intake was used to constrain the model as explained in Supplemental Methods. Shown are vitamin A masses in compartments 5, 6, and 7; dietary vitamin A input; and vitamin A disposal rate [calculated as L(10,6) × M(6)]. Model parameters are mean sojourn time (days) to retinol-binding protein (i.e., the time required for dietary vitamin A to be transported through the gastrointestinal tract, absorbed and incorporated into chylomicrons, cleared by the liver, and secreted into plasma as retinol bound to retinol-binding protein) and fractional transfer coefficients (the fraction of retinol in compartment J transferred to compartment I each day). For known values generated for the full model (Figure 2), mean sojourn time was calculated as the sum of the turnover times for compartments 1, 2, and 4 plus the delay time (days) in delay component 3 [i.e., L(2,1)^−1^ + L(3,2)^−1^ + DT(3) + L(5,4)^−1^] and as the delay time (days) in delay component 3 plus the turnover time in compartment 4 [i.e., DT(3) + L(5,4)^−1^] for the simplified model used for protocol 3 (Figure 2, inset). L(I,J), fractional transfer coefficient; M(I), vitamin A mass in compartment I; MST_RBP_, mean sojourn time to retinol-binding protein.

**FIGURE 3:**
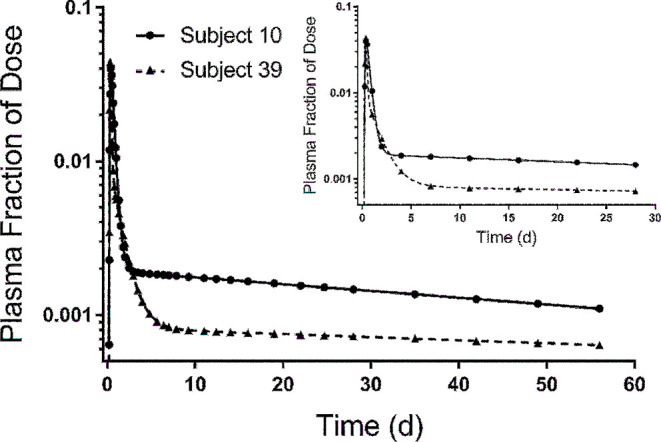
Model-simulated plasma retinol response data vs. time after ingestion of labeled retinyl acetate for 2 theoretical subjects. Shown are data for the fraction of dose simulated at all sampling times (*n* = 36) from 6 h to 56 d using parameters listed in Supplemental Table 1. For subject 39, we used the model (Figure 2) with both extravascular compartments (compartments 6 and 7); for subject 10, we used the model with only one compartment (compartment 6). The inset shows simulations for the same subjects using the reduced (super-child) sampling schedule (*n* = 11 samples collected from 6 h to 28 d).

Using the model shown in Figure 2 and the kinetic parameters listed in Supplemental Table 1, we simulated FD_p_ versus time for labeled retinol for each theoretical subject. Tracer response curves for 2 selected children are shown in Figure 3. Data for subject 10 were generated using the model with one extravascular compartment (compartment 6; Figure 2) that had a vitamin A mass (TBS) of 199 µmol; the model with 2 extravascular compartments (compartments 6 and 7) was used for subject 39, for whom vitamin A mass in compartment 6 (TBS) was 777 µmol and vitamin A mass in compartment 7 was 23 µmol. As shown by these 2 curves and as seen in plots generated for other subjects using parameters presented in Supplemental Table 1, plasma tracer responses followed previously observed patterns (12, 13), indicating an early absorptive phase followed by mixing of retinol into other pool(s) and subsequent recycling to plasma. Curves then transitioned into a terminal slope, reflecting the fractional catabolic rate for vitamin A in stores [i.e., FCR_TBS_ = L(10,6)].

For each theoretical subject, we also used the model to simulate time-variant values for the RID equation coefficients (*Fa* and *S*) from 6 h to 56 d. When we used those values at any given time in RID Equation *2*, along with each child's corresponding SA_p_, predictions of TBS were identical to the known values [i.e., M(6)] at all times. That is, by using the correct (model-calculated) values for the coefficients *Fa* and *S*, one can accurately calculate TBS at any time that SA_p_ is measured. This is shown in **Supplemental Figure 1** for subject 42 [also see Supplemental Methods for calculations equating TBS to M(6)].

### Results for extensive and reduced sampling schedules (protocols 1 and 2)

To establish proof-of-concept that population-based modeling provides accurate estimates for retinol kinetic parameters and state variables, including vitamin A TBS, we analyzed the extensive sampling data set (all 50 subjects at all 36 times; **Supplemental Figure 2**A) (protocol 1). As discussed in more detail in Supplemental Results, model-predicted population values for all kinetic parameters and state variables were within the ranges of the known values for the 50 theoretical children and were similar to the known group (geometric mean) values (**Supplemental Table 2**). Of particular note, population TBS [M(6)] predicted by the model (535 µmol) was within 1% of the geometric mean of the assigned values (538 µmol). Next, we applied the RID equation (Equation *2*) to predict TBS in individual subjects using the population model-predicted value for the composite coefficient *Fa* × *S* at 4 d and each subject's SA_p_ at that time. As shown in **Supplemental Table 3**, compared with the assigned values for each theoretical child, predictions of TBS at 4 d were within 25% of the known value for 78% of children, determined by calculating [(predicted TBS − assigned TBS) ÷ assigned TBS] × 100. Overall, these results established proof-of-concept that, when detailed kinetic data were used, the population-based approach accurately predicted group TBS, provided good estimates for population retinol kinetics, and predicted TBS for individual children reasonably well.

Then, to evaluate the approach with fewer sampling times (protocol 2), we performed a similar analysis using data for all subjects but only at times included in the reduced sampling schedule (28 rather than 56 d and 11 rather than 36 times; Supplemental Figure 2B). As was true for the analysis of protocol 1, population values for the model parameters and state variables (Supplemental Table 2) were predicted within the ranges of the known values and were comparable to the group mean values; TBS predicted by the model (537 µmol) was within 1% of the known group value (538 µmol). See Supplemental Results for more detailed presentation of results as well as additional analyses related to model evaluation. As was true for protocol 1, predictions of TBS at 4 d by RID (Equation *2*) were also within 25% of the known value for 78% of subjects (Supplemental Table 3). Results showed that the reduced sampling schedule provided enough data at the kinetically sensitive times that are needed to define the dynamics of the system, including a sufficient number of data points (4–5) to define the terminal slope once it was reached (between 7 and 10 d; shown in Supplemental Figure 2B for mean data and also in the Figure 3 inset for 2 selected subjects). On the basis of these results, we conclude that 28 d was an adequate time for tracer mixing and thus a sufficient study duration for these theoretical children.

### Results for super-child protocol (protocol 3)

**FIGURE 4:**
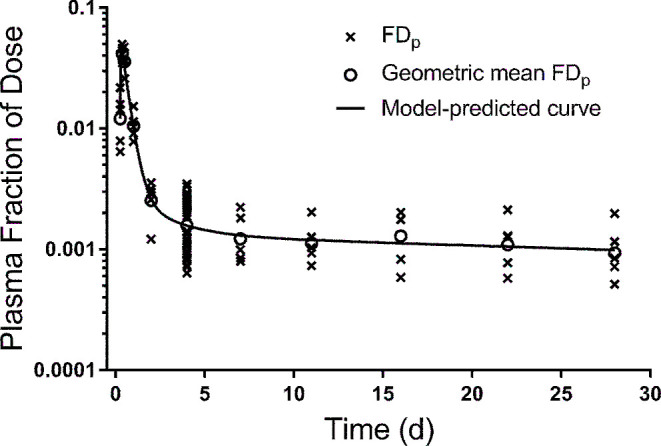
Model-simulated composite plasma retinol response data vs. time after ingestion of labeled retinyl acetate for 50 theoretical subjects generated using the super-child protocol. Shown are “observed” FD_p_ for individual subjects (50 subjects at 4 d and 5 randomly assigned children at each remaining time), the geometric mean FD_p_ at each time, and the model-calculated fit to the mean data for 1 of the 5 super-child data sets (protocol 3/scenario 5). FD_p_, fraction of dose in plasma.

Next, we evaluated the population-based approach using the design being implemented in ongoing super-child studies (the reduced sampling schedule used in protocol 2 but with only 2 samples from each child) using 5 population data sets (scenarios) as described in Methods. Figure 4 shows the data for scenario 5, showing observed FD_p_ versus time for individual subjects as well as the geometric FD_p_ calculated at each time and the model-calculated fit to the geometric mean data (see **Supplemental WinSAAM Deck**); **Supplemental Figure 3** shows analogous data for the other 4 scenarios. Note that the model with 2 extravascular compartments (compared with 1) did not significantly improve the fit to the mean data for protocol 3 based on an F-statistic. However, the former model provided a better fit on the basis of visual interpretation and was justified for all 5 scenarios because it improved predictions of both TBS (to within our evaluation criterion of 20% of the known group value) and model parameters for the group. Compared with the geometric mean and range of the known values for the group and similar to results for protocols 1 and 2, model-predicted values for state variables and kinetic parameters were within the ranges of the known values and were comparable to the known group values. Selected data for the 5 scenarios are shown in Table 1, and all parameters are listed in Supplemental Table 2. In addition, model-predicted values for parameters that are important for defining the system kinetics and for accurate predictions of TBS [M(6)] were adequately estimated as were dietary input [U(3)] and disposal rate (see Supplemental Results for additional results, including use of dietary intake data to constrain the model for one scenario). As shown in Table 2, for all 5 scenarios, values for TBS predicted by the population model were, on average, within 8% (range: 1–17%) of the known group mean value.

**TABLE 2:** Vitamin A TBS for theoretical children predicted by population modeling and by RID, with composite RID equation coefficients and outcome evaluation, for protocol 3^1^

	Scenario 1	Scenario 2	Scenario 3	Scenario 4	Scenario 5
Model-predicted TBS, µmol	445	534	489	573	505
RID-predicted TBS at 4 d, µmol					
Geometric mean	572	535	483	574	503
Range	68–2210	82–2658	74–2402	88–2851	77–2498
Model-predicted *Fa *× *S* at 4 d	0.69	0.83	0.75	0.89	0.78
Outcome evaluation, % of children					
within 25%	76	76	80	66^2^	78
within 50%	96	96	96	94	96
within 75%	100	100	100	100	100

1 Values are vitamin A TBS [M(6) in Figure 2] predicted by population modeling of the 5 data sets that included all 50 theoretical subjects on day 4 and 5 randomly selected subjects at each of the other 10 reduced sampling times (protocol 3/scenarios 1–5). Note that, when fitting scenario 4 data to the model in Figure 2 inset, the geometric mean dietary intake (4.1 µmol/d) was used to constrain L(10,6) (see Supplemental Methods for more details). Also shown are the geometric mean and range for TBS predicted by RID (Equation *2*; TBS = *Fa* × *S* × 1/SA_p_) at 4 d and population model-predicted values for the composite RID coefficient *Fa* × *S* at 4 d, calculated as F(6) × {[F(5) ÷ M(5)] ÷ [F(6) ÷ M(6)]}, where F(I) is tracer in compartment I at time *t* (4 d) and M(I) is vitamin A mass in compartment I. Compare these values for TBS with the geometric mean of the assigned values for all 50 theoretical children (538 µmol; range: 92–1904 µmol). Outcome criteria are percentage of children whose 4-d predicted TBS was within 25%, within 50%, or within 75% of the assigned value. *Fa*, fraction of dose of labeled vitamin A absorbed and found in the body's storage pool at time *t*; RID, retinol isotope dilution; *S*, ratio of retinol specific activity in plasma to that in the storage pool at time *t*; TBS, total body stores.

2 Below our evaluation criterion (predicted value was within 25% of the known value for ≥75% of children).

**FIGURE 5:**
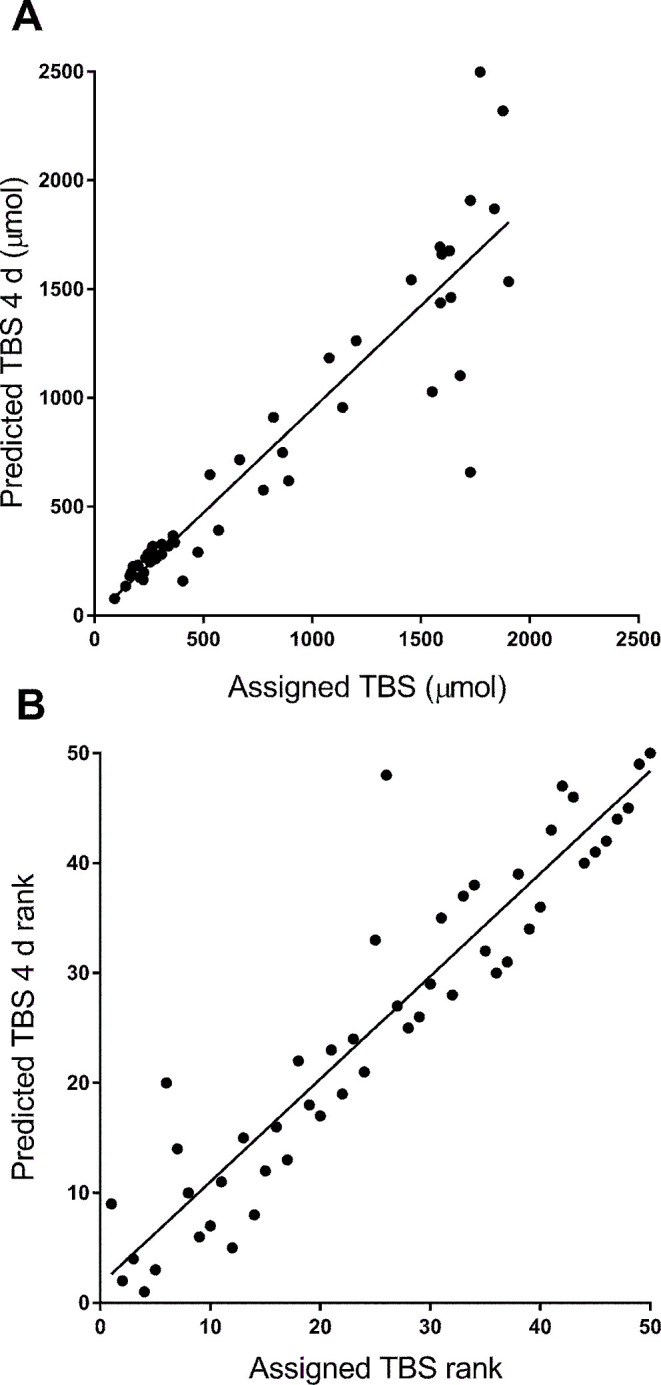
Assigned compared with predicted values for vitamin A TBS for 50 theoretical subjects (A) and rank-order of values (B). TBS was predicted using retinol isotope dilution (Equation *2*) at 4 d for 1 of the 5 super-child data sets (protocol 3/scenario 5); least-squares regression lines were *y* = 0.95*x* − 4.1 (*R*^2^ = 0.86, *P *< 0.0001) (A) and *y* = 0.93*x* + 1.7 (*R_s_*_ _= 0.93, *P *< 0.0001) (B). TBS, total body stores.

When we used the population model to calculate values for the composite coefficient *Fa* × *S*, values at 4 d for the 5 super-child data sets ranged from 0.69 to 0.89; these were similar to the geometric mean for the individual subjects (0.84) (Table 2). Using these values in the RID equation at 4 d, TBS predictions for 4 of the 5 scenarios met our criterion; that is, they were within 25% of the known value for ≥76% of subjects. For the remaining scenario (scenario 4), our criterion was not met; however, predictions were within 25% of the known value for 66% of children in the group. In fact, 4-d predicted TBS for all 5 scenarios were within 50% of the known value for >90% of subjects and estimates were within 75% of the known value for all theoretical children. For the 5 scenarios, the arithmetic mean ratio of 4-d predicted/known TBS ranged from 0.85 to 1.10, the ratio for individual subjects ranged from 0.34 to 1.61, and the range of TBS predicted at 4 d reflected the wide range in assigned values for TBS (Table 2). Figure 5A shows 4-d TBS predictions for scenario 5 compared with the assigned value for each theoretical child in the group (*R*^2^ = 0.86, *P *< 0.0001). Similar regressions for the other 4 scenarios were also strong and significant (data not shown). Although the absolute difference between the 4-d prediction and the assigned value was greater at higher TBS, the variation in the ratio of predicted/known values was similar across the range of TBS (**Supplemental Figure 4**). In addition, as expected, over all 5 scenarios, when we calculated the geometric mean for the individual TBS values predicted at 4 d, we found that, on average, the value was within 6% of the known value for the group (range: 1–10%). For all scenarios, as well as for protocols 1 and 2, when 4-d TBS predictions and assigned values were ranked, the rank-order correlation was strong and significant (*R_s_* = 0.93, *P* < 0.0001); these results are shown in Figure 5B for the data from scenario 5 used in Figure 5A and presented for protocols 1 and 2 in Supplemental Results.

We also noted that the difference between 4-d predictions of TBS and assigned values was due to variation in individual subject values for the composite coefficient *Fa* × *S* at 4 d (range: 0.55–2.0; mean: 0.84) compared with the population model-predicted value (Table 2). In addition, most (86%) of the variance in TBS predictions at 4 d was due to variance in the parameter measured experimentally (SA_p_) and used in the equation as 1/SA_p_; the remaining 14% was due to the composite coefficient *Fa* × *S*.

## Discussion

In the current work, we used theoretical data to validate a population-based (super-child) approach for investigating retinol kinetics and assessing vitamin A status in children. A population-based approach has been previously used in the vitamin A field by Haskell et al. (18) and Lopez-Teros et al. (14). When combined with modeling, as was done in the latter study, the super-child approach offers several advantages. This approach minimizes the number of blood samples required from each participant while, as our results show, accurately predicting population vitamin A TBS and also providing information on whole-body vitamin A kinetics for the group, data that are not provided when using assessment methods that do not include compartmental modeling. In addition, population-specific values for the RID composite coefficient (*Fa* × *S* in Equation *2*) can be obtained from the population model and used to improve individual subject TBS predictions at an appropriate time after dosing. Although population-based modeling is increasingly used in pharmacokinetics and is explicitly recommended by regulatory agencies for studies in children (15), the use of such methods for estimating TBS and retinol kinetics has, to our knowledge, not yet been validated for studying vitamin A, as we did here. Our strategy was to establish a physiologically based compartmental model using previously published models (12–14) and then populate that model with known values for retinol kinetic parameters and state variables to generate a database for 50 theoretical children. Our results for the 3 protocols tested (all subjects and an extensive sampling schedule, all subjects and a reduced sampling schedule, and a reduced number of subjects and sampling times) showed that the population-based approach estimated population retinol kinetics well (Table 1, Supplemental Table 2), accurately predicted population vitamin A TBS, and provided good estimates for individuals over a wide range of TBS (Table 2, Supplemental Table 3).

Although the current work was conducted for a group of theoretical children with high vitamin A intakes (658–4862 µg RAEs/d), reflecting anticipated conditions in ongoing field studies, we also applied the population-based method to mean data for a small group of theoretical children (*n* = 10) who had low to moderate vitamin A intakes (range: 234–585 µg/d) and low vitamin A stores (54–114 µmol) (unpublished observations JL Ford, MH Green, 2018). We used both protocol 1 and 2 sampling schedules for this group and found that TBS calculated by the population model (84 µmol) accurately predicted the geometric mean of the individual known values (85 µmol). In addition, when we applied the RID equation using the population model-predicted value for the composite coefficient *Fa* × *S* at 4 d (0.84) calculated for these subjects using protocol 2, predictions were within 25% of the known values for all 10 subjects; the arithmetic mean ratio of 4-d predicted/known TBS was 1.02 (range: 0.76–1.2). These results indicate that the super-child approach can provide accurate predictions of population TBS for levels of vitamin A status ranging from low/marginal to adequate/high and that it can predict TBS for individuals over this range of statuses reasonably well.

As part of our study, we investigated the potential impact of assignment of individuals to a second sampling time by analyzing 5 super-child data sets in which subjects were randomly assigned to the second time (protocol 3). We found, for all 5 data sets, that the composite plasma tracer response curve adequately represented the true central tendency for the group (Figure 4, Supplemental Figure 3) and that the model accurately predicted population TBS (Table 1). In previous studies that applied a super-child approach (14, 18), the design included the collection of 2 blood samples from each child and included 2–5 children at each sampling time; the sample size for one (18) was large (*n* = 107) and for the other (14) it was smaller (*n* = 15). Similar designs have been used for population-based pharmacokinetic modeling, in which 2–3 samples are collected from each individual, often from a cohort of ∼50 participants (29). Our results, for a group of 50 individuals, indicate that collecting 2 blood samples/child, with all subjects sampled at one specific time (e.g., 4 d) and 5 children sampled at each of the remaining times, provided data that adequately reflected the central tendency for group mean TBS and retinol kinetics and that can also be used to predict TBS in individual subjects. Overall, these results will help public health nutritionists better design future studies aimed at assessing vitamin A TBS and retinol kinetics in children.

One reason that RID as traditionally applied generally works better for groups than individuals (3) is that applications of RID equations have, until recently (21), typically used the same values for the coefficients across individuals and populations. Consistent with the hypothesis that the accuracy of RID predictions for individuals could be improved by including values for the coefficients that have been calculated for the population being studied, we recently showed (21) that, when the model-predicted group mean values for the coefficients were used at 4 or 5 d postdosing, RID, in fact, predicted TBS in individuals quite accurately. Here, we used modeling results to calculate a population-specific value for the time-variant composite coefficient *Fa* ×* S* in the RID equation (Equation *2*) and applied the 4-d value to predict TBS in individual subjects (Table 2, Supplemental Table 3). A similar technique was used in our work with Lopez-Teros et al. (14), in which population values for *Fa* ×* S* were used in the RID equation with plasma tracer data for 7 individuals sampled between 3 and 6 d after dosing. In that study, we found excellent agreement between equation-predicted TBS for individuals (geometric mean: 816 µmol; range: 689–1141 µmol) and the model-predicted value for those individuals (823 µmol). Our current analysis suggests that the predicted range for that group of children is reasonably accurate. It is important to note that, by applying model-based compartmental analysis in combination with a super-child approach, population values can be calculated for RID equation coefficients at any time; thus, TBS can be calculated for individual subjects at any appropriate time (e.g., 4–14 d) that blood was sampled.

For the group of young adults studied in the work of Green et al. (21), we reported that predictions by the RID equation were most sensitive to the measured variable (SA_p_) when the CV for the composite coefficient *Fa* × *S* was lowest, at which time TBS predictions were most accurate; this was at 4 or 5 d after dosing (CV = 15%). At those times, >90% of the variance in TBS predictions was accounted for by variance in 1/SA_p_. In the current study, the CV for *Fa* × *S* at 4 d was higher (33%), largely due to the influence of the second extravascular pool (compartment 7; Figure 2), which was present for approximately half of our subjects. Specifically, the CV at 4 d was low (10%) in subjects with only one extravascular pool (storage compartment 6), similar to results for the adults studied by Green et al. (21), and it was much higher (33%) for subjects with 2 extravascular compartments. However, for all 50 subjects, TBS was still highly sensitive to 1/SA_p_ at 4 d, accounting for 86% of the variance in TBS. Focusing on the super-child sampling times, the CV for the composite coefficient *Fa* × *S* was lowest at 7 d (12%) and, at that time, the influence of the second extravascular compartment was minimal. In fact, if we apply protocol 2’s population value for *Fa* × *S* at 7 d, individual predictions of TBS were closer to the known values than those at 4 d (within 25% of the assigned value for 94% of subjects at 7 d compared with 78% of subjects at 4 d) and the arithmetic mean ratio of 7-d predicted/known TBS was 0.99 (range: 0.67–1.26). Interestingly, when we retrospectively applied Equation *2* to 7-d data for the adults studied by Green et al. (21), we found that predictions at 7 d were not significantly different from values predicted at 4 d (*P* = 0.24). Overall, to obtain the best predictions of individual subject TBS when using a population value for *Fa* ×* S*, researchers should apply Equation *2* at a time when variance in the composite coefficient is lowest for the population. Our current results suggest that, in future super-child studies, it may be optimal to sample all children at 7 rather than at 4 d after dosing.

Our decision to use a model with one extravascular compartment for some of our theoretical subjects compared with 2 for others was based on previous research (11–14), but it introduced complexities into the current analysis. Although the one relevant study conducted to date in children required a model with 2 extravascular compartments (14), researchers will not know before the start of an experiment whether a model with >1 extravascular compartments will be required. As additional vitamin A modeling studies are conducted in children, it may be found that 2 extravascular compartments are generally required for this age group.

In conclusion, the population-based (super-child) approach provides a more feasible design for studying retinol kinetics and vitamin A status in children than do traditional (nonpopulation) methods because it requires collection of a minimal number of blood samples from each child. Using the super-child protocol and compartmental analysis, we confirmed our hypothesis that retinol kinetic parameters and total body vitamin A stores can be estimated for a given group of children with reasonable accuracy. In addition, population-specific values for the RID equation's composite coefficient *Fa* × *S* can be obtained and used to predict TBS for individuals relatively soon after the administration of the stable isotope dose; current results indicate that 7 d may be the optimal time. By conducting super-child studies in various populations, researchers will be able to refine estimates for the composite coefficient, thereby improving the accuracy of isotope dilution predictions of vitamin A stores in individuals, as well as increasing understanding of the dynamics of retinol metabolism in children. Overall, our results indicate that this approach should be useful for assessing vitamin A status in children over a wide range, providing a method that can be applied to evaluate the efficacy and potential risk associated with vitamin A intervention programs.

## Supplementary Material

Supplement File

## Notes

Supported by the Bill & Melinda Gates Foundation (project OPP1115464) and the College of Health and Human Development, The Pennsylvania State University.

Author disclosures: JLF, JBG, and MHG, no conflicts of interest.

Supplemental Methods, Supplemental Results, Supplemental Tables 1–3, Supplemental Figures 1–5, Supplemental WinSAAM Deck, and Supplemental References are available from the “Supplementary data” link in the online posting of the article and from the same link in the online table of contents at https://academic.oup.com/cdn/.

Abbreviations used:

FD_p_: fraction of administered dose in plasma
RAE: retinol activity equivalent
RID: retinol isotope dilution
SA_p_: specific activity of retinol in plasma (fraction of oral tracer dose/mass of tracer plus tracee)
TBS: total body stores

## References

[1] WHO. Guideline: vitamin A supplementation in infants and children 6–59 months of age. Geneva (Switzerland): WHO; 2011. 24575452

[2] Tanumihardjo SA, Mokhtar N, Haskell MJ, Brown KH Assessing the safety of vitamin A delivered through large-scale intervention programs: workshop report on setting the research agenda. Food Nutr Bull 2016;37:S63–74. 26893060 10.1177/0379572116630480

[3] Lietz G, Furr HC, Gannon BM, Green MH, Haskell M, Lopez-Teros V, Novotny JA, Palmer AC, Russell RM, Tanumihardjo SA Current capabilities and limitations of stable isotope techniques and applied mathematical equations in determining whole-body vitamin A status. Food Nutr Bull 2016;37:S87–103. 27053491 10.1177/0379572116630642

[4] Tanumihardjo SA, Russell RM, Stephensen CB, Gannon BM, Craft NE, Haskell MJ, Lietz G, Schulze K, Raiten DJ Biomarkers of Nutrition for Development (BOND)—vitamin A review. J Nutr 2016;146(Suppl):1816S–48. 27511929 10.3945/jn.115.229708 PMC4997277

[5] Green MH Evaluation of the “Olson Equation,” an isotope dilution method for estimating vitamin A stores. Int J Vitam Nutr Res 2014;84(Suppl 1):9–15. 25537101 10.1024/0300-9831/a000181

[6] Cifelli CJ, Green MH, Green JB Use of model-based compartmental analysis to study vitamin A kinetics and metabolism. Vitam Horm 2007;75:161–95. 17368316 10.1016/S0083-6729(06)75007-5

[7] Green MH, Uhl L, Green JB A multicompartmental model of vitamin A kinetics in rats with marginal liver vitamin A stores. J Lipid Res 1985;26:806–18. 4040952

[8] Lewis KC, Green MH, Green JB, Zech LA Retinol metabolism in rats with low vitamin A status: a compartmental model. J Lipid Res 1990;31:1535–48. 2246607

[9] Tan L, Green MH, Ross AC Vitamin A kinetics in neonatal rats vs. adult rats: comparisons from model-based compartmental analysis. J Nutr 2015;145:403–10. 25540407 10.3945/jn.114.204065 PMC4336526

[10] Escaron AL, Green MH, Howe JA, Tanumihardjo SA Mathematical modeling of serum ^13^C-retinol in captive rhesus moneys provides new insights on hypervitaminosis A. J Nutr 2009;139:2000–6. 19710158 10.3945/jn.109.111922 PMC2744618

[11] von Reinersdorff D, Green MH, Green JB Development of a compartmental model describing the dynamics of vitamin A metabolism in men. Adv Exp Biol Med 1998;445:207–23. 10.1007/978-1-4899-1959-5_13 9781391

[12] Cifelli CJ, Green JB, Wang Z, Yin S, Russell RM, Tang G, Green MH Kinetic analysis shows that vitamin A disposal rate in humans is positively correlated with vitamin A stores. J Nutr 2008;138:971–7. 18424609 10.1093/jn/138.5.971

[13] Green MH, Ford JL, Oxley A, Green JB, Park H, Berry P, Boddy AV, Lietz G Plasma retinol kinetics and β-carotene bioefficacy are quantified by model-based compartmental analysis in healthy young adults with low vitamin A stores. J Nutr 2016;142:2129–36. 10.3945/jn.116.233486 PMC5037873 27511941

[14] Lopez-Teros V, Ford JL, Green MH, Tang G, Grusak MA, Quihui-Cota L, Muzhingi T, Paz-Cassini M, Astiazaran-Garcia H Use of a “super-child” approach to assess the vitamin A equivalence of *Moringa oleifera* leaves, develop a compartmental model for vitamin A kinetics, and estimate vitamin A total body stores in young Mexican children. J Nutr 2017;147:2356–63. 28931584 10.3945/jn.117.256974

[15] Meibohm B, Laer S, Panetta JC, Barret JS Population pharmacokinetic studies in pediatrics: issues in design and analysis. AAPS J 2005;7:E475–8. 16353925 10.1208/aapsj070248 PMC2750985

[16] Batchelor HK, Marriot JF Pediatric pharmacokinetics: key considerations. Br J Clin Pharmacol 2015;79:395–404. 25855821 10.1111/bcp.12267 PMC4345950

[17] Cifelli CJ, Green JB, Green MH Dietary retinoic acid alters vitamin A kinetics in both the whole body and in specific organs of rats with low vitamin A status. J Nutr 2005;135:746–52. 15795428 10.1093/jn/135.4.746

[18] Haskell MJ, Lembcke JL, Salazar M, Green MH, Peerson JM, Brown KH Population-based plasma kinetics of an oral dose of [^2^H_4_]retinyl acetate among preschool-aged, Peruvian children. Am J Clin Nutr 2003;77:681–6. 12600861 10.1093/ajcn/77.3.681

[19] Wastney ME, Patterson BH, Linares OA, Greif PC, Boston RC WinSAAM. In: Investigating biological systems using modeling: strategies and software. San Diego (CA): Academic Press; 1999 p. 95–138.

[20] Stefanovski D, Moate PJ, Boston RC WinSAAM: a Windows-based compartmental modeling system. Metabolism 2003;52:1153–66. 14506622 10.1016/s0026-0495(03)00144-6

[21] Green MH, Ford JL, Green JB, Berry P, Boddy AV, Oxley A, Lietz G A retinol isotope dilution equation predicts both group and individual total body vitamin A stores in adults based on data from an early postdosing blood sample. J Nutr 2016;146:2137–42. 27511937 10.3945/jn.116.233676 PMC5037874

[22] Green MH, Ford JL, Green JB Retinol isotope dilution is applied during restriction of vitamin A intake to predict individual subject total body vitamin A stores at isotopic equilibrium. J Nutr 2016;146:2407–11. 27683870 10.3945/jn.116.238899

[23] Ford JL, Green JB, Lietz G, Oxley A, Green MH A simple plasma retinol isotope ratio method for estimating β-carotene relative bioefficacy in humans: validation with the use of model-based compartmental analysis. J Nutr 2017;147:1806–14. 28747484 10.3945/jn.117.252361

[24] Berman M, Weiss MF SAAM manual. Washington (DC): US Government Printing Office; 1978 DHEW Publication No.: (NIH) 78-180.

[25] Park H. Model-based compartmental analysis of the kinetics of retinol and β-carotene in humans: statistical considerations in designing and building models for retinol plus expanded models for β-carotene [dissertation]. State College (PA): The Pennsylvania State University; 2011.

[26] Landaw EM, DiStefano JJ Multiexponential, multicompartmental, and noncompartmental modeling. II. Data analysis and statistical considerations. Am J Physiol 1984;246:R665–77. 6720989 10.1152/ajpregu.1984.246.5.R665

[27] Furr HC, Green MH, Haskell M, Mokhtar N, Nestel P, Newton S, Ribaya-Mercado JD, Tang G, Tanumihardjo S, Wasantwisut E Stable isotope dilution techniques for assessing vitamin A status and bioefficacy of provitamin A carotenoids in humans. Public Health Nutr 2005;8:596–607. 16236189 10.1079/phn2004715

[28] Gannon BM, Tanumihardjo SA Comparisons among equations used for retinol isotope dilution in the assessment of total body stores and total liver reserves. J Nutr 2015;145:847–54. 25809683 10.3945/jn.114.208132 PMC6619684

[29] Anderson BJ, Allegaert K Population clinical pharmacology of children: general principles. Eur J Pediatr 2006;165:741–6. 16807730 10.1007/s00431-006-0188-y

