# Supplementary material for: A Population-Based (Super-Child) Approach for Predicting Vitamin A Total Body Stores and Retinol Kinetics in Children Is Validated by the Application of Model-Based Compartmental Analysis to Theoretical Data
Source: Curr Dev Nutr. 2018 Nov 24;2(11):nzy071. doi: 10.1093/cdn/nzy071 (PMC6252344; doi:10.1093/cdn/nzy071)
Supplement: Supplement File [file nzy071_supplement_file.pdf]

Supplemental Methods

Generation of theoretical data for individuals. In overview, in order to generate known values for state variables and model parameters, we first established physiologically-reasonable ranges for the specified variables and parameters based on information in the literature; then, we generated 50 values within each range using RStudio (Supplemental Reference 1) and assigned a value to each theoretical child, either randomly or using systematic grouping. Assigned values for the appropriate variables and parameters were then used to calculate known values for the remaining kinetic parameters, as follows.

Beginning with vitamin A TBS, we generated 50 values within a physiologically-reasonable range (90 – 2,000 µmol) and we assigned each value to a theoretical child. We established ranges for plasma retinol concentration [0.75 – 2.4 µmol/L (Supplemental References 2 and 3)] and for body weight; for the latter, we used WHO weight-by-age charts for children ages 6 mo to 5 y [8 – 24 kg (Supplemental Reference 4)]. Individual values for plasma retinol concentration and body weight were generated and assigned using systematic grouping as follows: assigned values for TBS [M(6), or the mass (µmol) of vitamin A in storage compartment 6; Figure 2] were ranked from low to high and then grouped. In the case of plasma retinol concentration, after values for TBS were grouped as lower (90 to 400 µmol; n=25) or higher (405 to 2,000 µmol; n=25), values for plasma retinol were generated between the range of 0.75 and 1.4 µmol/L for the lower TBS group and between 1.3 and 2.4 µmol/L for the higher TBS group; values for retinol concentration were then randomly paired with a value for TBS within the appropriate group. For body weight, values were generated within specified ranges determined for 8 groupings of TBS and randomly paired with a value for TBS within that group. That is, since infants are born with essentially no vitamin A stores and as they age, TBS increases (Supplemental Reference 5), body weight was associated with age and TBS in generating the current data. For example, for values of TBS between 200 and 300 µmol, values for body weight were generated within a range of 8 and 14 kg; for a range of TBS between 1,500 and 1,700 µmol, values for body weight were generated between 16 and 20 kg. Following assignment of plasma retinol concentration and body weight, values for plasma retinol pool size [µmol; M(5) in the model], which are needed for steady state calculations in WinSAAM, were calculated for each child as plasma retinol concentration (µmol/L) × body weight (kg) × estimated plasma volume (L/kg)], where plasma volume was estimated as 0.05 L/kg body weight (Supplemental Reference 6).

To simulate "observed tracer data" versus time after ingestion of labeled retinyl acetate for each theoretical child, we needed to first generate known values for each of the kinetic parameters shown in model Figure 2; these included fractional transfer coefficients [L(I,J)s, or the fractional transfer of retinol in compartment J to compartment I each day] and a delay element [DT(3), or the time spent in delay component 3]. We used information in the literature (12 – 14) to establish physiologically-reasonable ranges for the model parameters listed in the next sentence; then 50 hypothetical values for each parameter were randomly generated in RStudio (Supplemental Reference 1) and each value was randomly assigned to a theoretical child. For L(2,1), the range was 10 – 20 d^{-1}; for DT(3), 0.15 – 0.25 d (3.6 – 6 h); for L(4,3), 0.7 – 0.9 d^{-1}; for L(5,4), 1 – 15 d^{-1}; and for L(6,5), the range was 20 – 40 d^{-1}. Loss of unabsorbed tracer [L(0,3)] was calculated as 1 minus L(4,3), where L(4,3), the fractional absorption efficiency of the labeled retinyl acetate dose, was varied between 0.7 and 0.9 (Supplemental Reference 2).

Values for L(10,6) [the fractional catabolic rate of vitamin A in stores (FCR_{TBS})] were generated within a range of 0.002 – 0.022 d^{-1} (14, 18, 28) and assigned using systematic grouping so that values for the derived parameters, vitamin A disposal rate [DR; R(10,6) (µmol/d) or the rate of vitamin A irreversible loss from compartment 6] and dietary vitamin A input [U(1), µmol/d], were reasonable. First, assigned values for TBS [M(6)] were ranked from

low to high and then partitioned into 8 groups. Values for L(10,6) were generated within the specified range for each grouping of TBS and randomly paired with a value for M(6) within that group. Then, assuming a steady state (vitamin A input = vitamin A output), vitamin A DR can be calculated as R(10,6) = L(10,6) × M(6) and dietary vitamin A input as U(1) = R(10,6) ÷ fractional absorption efficiency [L(4,3)]. Ranges for L(10,6) were determined for each grouping of M(6) to obtain reasonable values for DR that translated to intakes between 2.1 and 17.5 μmol/d (600 – 5,000 μg RAE/d). For example, for values of M(6) between 200 and 300 μmol, values for L(10,6) were generated within a range of 0.009 and 0.012 d^{-1}, resulting in values for R(10,6) that ranged from 2.4 to 2.7 μmol/d and for U(1) that ranged from 2.9 to 3.8 μmol/d; for a range of M(6) between 1,500 and 1,700 μmol, values for L(10,6) were generated within a range of 0.002 and 0.005 d^{-1}, resulting in values for R(10,6) from 3.4 to 8.4 μmol/d and for U(1) from 3.9 to 11 μmol/d.

Then, we used the preassigned values for L(6,5) and L(10,6), along with steady state compartment masses for plasma retinol [M(5)] and the storage compartment [M(6)], to calculate L(5,6), or recycling of tracer to plasma from the storage compartment. In a steady state, R(I,J) (μmol retinol in compartment J transferred to compartment I each day) is calculated as R(I,J) = L(I,J) × M(J). Thus, L(5,6) = R(5,6) ÷ M(6), where R(5,6) = R(6,5) – R(10,6), R(6,5) = L(6,5) × M(5), and R(10,6) is calculated as described in the preceding paragraph.

For theoretical children whose data were simulated using two extravascular compartments (compartments 6 and 7), values for M(7) (μmol of vitamin A in compartment 7) were randomly generated between 1 and 12% of the value assigned for M(6), creating values for M(7) that ranged from 5 – 38 μmol. To obtain hypothetical values for L(7,5) for each child with 2 extravascular compartments, we randomly generated and assigned values for L(5,5), or the fraction of retinol that exits compartment 5 each day, within a range of 20 – 90 d^{-1}. Note that, for subjects with only 1 extravascular compartment (compartment 6), L(5,5) = L(6,5), whereas for subjects with 2 extravascular compartments, L(5,5) = L(6,5) + L(7,5). Thus, for the latter subjects, we calculated L(7,5) = L(5,5) – L(6,5); values for L(5,7) were calculated as R(5,7) ÷ M(7), where R(5,7) = R(7,5) because there is no loss from compartment 7, and R(7,5) = L(7,5) × M(5).

Next, in order to generate detailed plasma retinol isotope response data versus time after ingestion of labeled retinyl acetate for each theoretical child ("observed data"), times were first generated by a geometric progression (Supplemental Reference 7) as follows: $T_{i+1} = T_i\,(T_N \div T_1)^{[1 \div (n-1)]}$, where T_N is the final sampling time (56 d), T_1 is the initial sampling time (1 h), T_i is the current data point, T_{i+1} is the next time point, and *n* is the total number of data points (n=45); several times (n=3) were added and several were adjusted to include the 11 times being used in the super-child sampling schedule that is currently being implemented in the ongoing studies (6, 9, 12 h and 1, 2, 4, 7, 11, 16, 22, 28 d). A study duration of 56 d was chosen based on the work of Tang et al. (Supplemental Reference 8) and initial model simulations that showed that mixing of tracer with the body pool of vitamin A had occurred well before 56 d and therefore the system fractional catabolic rate was adequately defined. Next, for each subject, tracer was introduced into compartment 1 and the initial condition was set equal to 1, known values for model parameters were fixed, and data (as fraction of dose in plasma; FD_p) were simulated versus time using WinSAAM (19, 20). Note that, due to differences in the assigned values for DT(3), when data were simulated, the first time that labeled retinol was observed in plasma for all of the subjects was 6 h; thus, values for FD_p were actually generated from 6 h to 56 d (n=36).

Prediction of vitamin A TBS by retinol isotope dilution. The retinol isotope dilution equation, presented by Green et al. (21) and shown below, was used to predict vitamin A TBS:

$$\text{TBS } (\mu\text{mol}) = Fa \times S \times 1/SA_p$$

where *Fa* is the fraction of dose (FD) of labeled vitamin A absorbed and found in the body's storage pool at time *t* [FD in model compartment 6; FD(6)]; *S*, the ratio of retinol specific activity in plasma to that in the storage pool at time *t*, was calculated from the model as [FD(5) ÷ M(5)] ÷ [FD(6) ÷ M(6)]; and SA_p is plasma retinol specific activity at time *t* and was calculated as FD_p ÷ plasma retinol pool size (µmol) [i.e., $FD(5)_t \div M(5)_t$]. Thus, this equation can be restated mathematically as follows:

$$TBS = FD(6) \times \{[FD(5) \div M(5)] \div [FD(6) \div M(6)]\} \times \{1 \div [FD(5) \div M(5)]\} = M(6).$$

Use of dietary intake data to improve model predictions. In the case of 1 of the 5 sets of randomly-generated data designed to reflect ongoing super-child studies (protocol 3 / scenario 4), we found that, when iterations were performed using weighted nonlinear regression analysis, the value for L(10,6) (Figure 2) (i.e., FCR_{TBS}) converged to zero because the terminal slope of the tracer response curve was shallow. As suggested by Lopez-Teros et al. (14), we constrained L(10,6) to a physiologically-reasonable (non-zero) value based on the geometric mean dietary vitamin A intake for the group (4.1 µmol/d). To do this, we assumed a steady state (i.e., vitamin A input = vitamin A output and thus dietary vitamin A intake × fractional absorption efficiency = DR). The value for L(10,6) was manually adjusted until the model predicted the correct DR; then, L(10,6) was fixed at that value and the iteration process was repeated for this scenario to obtain the final model fit and parameter estimates. Note that, because for this theoretical analysis, dietary intake data were known, we fixed the value for L(10,6). However, when analyzing data collected from field studies, a standard deviation should be included as a constraint in WinSAAM to allow for small changes in the value of L(10,6) when iterations are performed.

Overall, this process shows that, in certain cases, dietary vitamin A intake can be helpful in increasing confidence in model predictions of TBS and kinetic parameters. Dietary data can be used as a "known" input into the model and could also be useful for confirming intake values predicted by the model and thereby improve the robustness of a model. Thus, for future studies assessing vitamin A status, including those using compartmental analysis, it is suggested that researchers collect reliable data on vitamin A intake; several available methods are described in (Supplemental Reference 9).

Supplemental Results

Detailed results for extensive sampling schedule (protocol 1). We first applied population-based modeling to the extensive sampling dataset (protocol 1), which included all 50 theoretical subjects at all 36 times from 6 h to 56 d, and estimated group mean values for retinol kinetic parameters and state variables, including vitamin A TBS. **Supplemental Figure 2A** shows geometric mean FD_p calculated at each time and the fit to the data using the 7 compartment model shown in Figure 2. Note that the model with 2 extravascular compartments (versus 1) provided both a significantly better fit to the mean data for the extensive sampling dataset as determined by an F-statistic and also gave a more accurate estimate for population TBS. As indicated in **Supplemental Table 2**, in which the modeling results are compared with the geometric means and ranges of the known values for the 50 theoretical children, all model-predicted values were within the ranges for the known values and were similar to the known group mean values; note that comparisons were made for the early kinetics (until retinol tracer reaches plasma compartment 5) using mean sojourn time to retinol binding protein [MST_{RBP}, or the time required for dietary vitamin A to be transported through the gastrointestinal tract, absorbed and incorporated into chylomicrons, cleared by the liver, and secreted into plasma as retinol bound to retinol binding protein]. TBS predicted by the population model (535 µmol) was within 1% of the known group value (538 µmol). Model parameters that are critical for defining the system kinetics and for accurately estimating TBS [L(6,5), L(5,6) and L(10,6); Figure 2] were predicted within 12% of the mean values for L(6,5) and L(5,6), and within 28% for L(10,6); dietary vitamin A intake and DR were predicted within 26 and 27% of the known group values, respectively. These results show that, for the extensive dataset, the population-based modeling approach accurately predicted group mean TBS and did a good job estimating population retinol kinetics.

Then, we used protocol 1's population-model predicted value for the composite RID equation coefficient $Fa \times S$ at 4 d (0.81) and we applied that value in Equation *2* with each child's SA_p at 4 d to predict individual values for TBS. As shown in **Supplemental Table 3**, predictions of TBS at 4 d for individual subjects met our evaluation criterion and were within 25% of the known value for 78% of children. In fact, 4 d predictions were within 50% of the known value for 96% of subjects and within 75% of the known value for all theoretical children. The arithmetic mean ratio of 4 d predicted / known TBS was 1.0 (range, 0.40 – 1.46) and the range of TBS predicted at 4 d reflected the wide range in known values for TBS. When 4 d predictions and assigned values were ranked (data not shown), the rank-order correlation was $R_s = 0.93$ ($P < 0.0001$). In addition, as expected, when we calculated the geometric mean for the individual TBS values predicted at 4 d, we found that the value was close to (within 3% of) the known group value (538 µmol). Also note that the difference between 4 d predictions of TBS and assigned values reflected the wide range in individual subject values for the composite coefficient $Fa \times S$ at 4 d (0.55 – 2.0; mean, 0.84). This is not surprising because some degree of variation is expected when a population value for the composite coefficient is used in Equation *2* to predict individual values for TBS.

Detailed results for reduced sampling schedule (protocol 2). Then, we did a similar analysis using population-based modeling and a reduced sampling dataset that included all subjects at reduced sampling times (11 times from 6 h to 28 d). Supplemental Figure 2B shows the fit to the simplified model (Figure 2 inset) of protocol 2's data for geometric mean FD_p versus time; note that, as for protocol 1, 2 extravascular compartments provided a statistically better fit to the mean data (see next section) and a more accurate prediction of group mean TBS. As we found for protocol 1 and is shown in Supplemental Table 2, population model-predicted values for kinetic parameters and state variables were within the ranges for the known values and were

comparable to the known group values. Specifically, population TBS [M(6)] was within 1% of the known group value (537 versus 538 µmol); model-predicted values for L(6,5) and L(5,6) (Figure 2) were within 6% of the known group values and L(10,6) was within 22%; and dietary intake and DR were within 20 and 22% of the mean values, respectively (Supplemental Table 2). Importantly, our results indicate that the reduced sampling schedule provided data at kinetically-sensitive times on the plasma retinol tracer response curve (i.e., provided data that were adequate to identify the model parameters). Also, while longer studies are preferred for modeling vitamin A kinetics in humans (e.g., at least 56 d in adults), for our current theoretical analysis, 28 d was sufficient to adequately define the true terminal slope of the tracer response curve, which is necessary for correctly estimating FCR_{TBS}, and thus vitamin A DR, as well as TBS.

Then, as we had done for analysis of protocol 1, we applied RID Equation *2* using the population model-predicted composite coefficient $Fa \times S$ at 4 d (0.84) with each subject's SA_p at that time. Predictions of TBS for individual subjects met our evaluation criterion and were within 25% of the assigned value for 78% of subjects. In fact, 4 d predicted TBS were within 50% of the known value for 94% of subjects and within 75% of the known value for 100% of children. The arithmetic mean ratio of 4 d predicted / known TBS was 1.04 (range, 0.41 – 1.52), reflecting the wide range in individual subject values for $Fa \times S$ at 4 d. In addition, the range of TBS predicted at 4 d reflected the wide range in assigned values for TBS (Supplemental Table 3). When 4 d predictions and known values were ranked, the rank correlation was identical to the results presented above for protocol 1. In addition, geometric mean 4 d TBS was within 1% of the known group value.

Analysis of subjects with 1 versus 2 extravascular compartments. As described in Methods, theoretical data for 50 hypothetical children were generated using a model with either 1 or 2 extravascular compartment(s). Even though a model with 2 extravascular compartments provided a statistically better fit to the geometric mean data for both protocols 1 and 2, we wanted to determine whether that was also true for the subset of 23 subjects whose data were generated with only 1 extravascular compartment. We first used the model with 1 extravascular compartment (compartment 6; Figure 2) to fit population data (i.e., geometric mean FD_p) using protocol 2 for those 23 subjects and found that TBS predicted by the population model was within 4% of the known group value (578 µmol versus 555 µmol). Similarly, when mean data for the subgroup of 27 subjects with 2 extravascular pools (compartments 6 and 7) were fit using the model with 2 extravascular compartments, population model-predicted TBS was within 1% of the known value (530 versus 525 µmol).

We also applied Equation *2*, with the population value for $Fa \times S$ at 4 d predicted using the model with 1 extravascular compartment (0.71) along with the appropriate SA_p at 4 d, for the 23 subjects with 1 extravascular pool. Predicted TBS was within 25% of the known value for 96% (22 of 23) of the subjects; the arithmetic mean ratio of 4 d predicted / known TBS was 1.01 (range, 0.79 – 1.28). In addition, when we did similar calculations for the subgroup with 2 extravascular compartments, for whom the population value for $Fa \times S$ at 4 d predicted using the model with 2 extravascular compartments was 1.0, TBS predicted using Equation *2* was within 25% of the known value for 78% (21 of 27) of subjects; the arithmetic mean ratio of 4 d predicted / known TBS was 1.07 (range, 0.49 – 1.49). **Supplemental Figure 5A** shows these 4 d predictions for subjects with 1 versus 2 extravascular compartments compared to the assigned value for each theoretical child (R^2 = 0.97 and 0.87, respectively; $P < 0.0001$ for both). For comparison, Supplemental Figure 5B shows predicted TBS at 4 d compared to the assigned value for each theoretical child for protocol 2, when all 50 children were included and population mean data were fit to the model with 2 extravascular compartments. These results indicate that TBS predictions at 4 d were more accurate for subjects with 1 extravascular compartment

compared to subjects with 2. In addition, predictions at 4 d were more accurate when we used the composite coefficient derived from the model with the same number of extravascular compartments as were used to generate the data for a given subject.

Additional results for super-child protocol (protocol 3). As shown in Supplemental Table 2, model-predicted values for L(6,5) and L(5,6) (or the fractional transfer of retinol to stores from plasma and the recycling of retinol back to plasma from stores, respectively; Figure 2) for all 5 scenarios in protocol 3 were close to the mean values (within 50%). For L(10,6) (FCR_{TBS}), model predictions were close to the mean values for a majority of the 5 scenarios tested; this was also true for dietary intake [U(3)] and DR, which are both mathematically associated with FCR_{TBS} [i.e., DR = L(10,6) × M(6) and U(3) = DR / fractional absorption efficiency]. It is not surprising that, in some cases, the value predicted for L(10,6) (FCR_{TBS}) was higher than the mean because of the reduced subject numbers at each (non-4 d) time and the random assignment of subjects at each time. This parameter is especially sensitive to data at later times (7 – 28 d) which are needed to adequately define the terminal slope. In spite of the higher predicted FCR_{TBS} for these scenarios, the model adequately predicted the group mean TBS (within 20% of the known group value).

Preliminary analysis of positive vitamin A balance on predictions of TBS by retinol isotope dilution. Presumably, chronic consumption of excessive amounts of vitamin A results in positive vitamin A balance (i.e., input is greater than output). Here, we assigned relatively high vitamin A intakes for our subjects (658 – 4,862 µg RAE/d) and because we hypothesized that subjects had adapted to these chronic high intakes (i.e., adaptive preservation or an increase in degradative utilization to balance intake) (Supplemental References 10 and 11) prior to the start of the study, we used a model (Figure 2) that assumed a steady state (input equals output). To investigate the impact on RID results of positive balance (i.e., if subjects had not adapted to these high intakes), we used an approach similar to that described by Ford et al. (Supplemental Reference 12). For this preliminary analysis, we chose 3 of our theoretical children to represent a range of conditions for TBS, vitamin A intake, and retinol kinetics (Supplemental Table 1; subjects 13, 27 and 49) and simulated the non-steady state condition of positive vitamin A balance assuming that 400 µg RAE/d (approximate RDA for this age group) would be the steady state intake for these subjects. Although we found that continuous excess intake resulted in a higher value for $Fa \times S$ compared to the steady state value at any given time and also that the magnitude of this difference increased with time, the impact on SA_p was minimal at all times over the 56 d. When the steady state value for $Fa \times S$ was used in the RID equation with SA_p simulated during positive balance, TBS predicted at 4 d were within 1 to 2.5% of the assigned value for the 3 subjects. However, the impact of positive balance on predicted TBS gradually increased with time and this effect was greater for the subject with lower stores. Thus, it is important to sample early when the effect on RID predictions is minimal.

SUPPLEMENTAL TABLE 1 Known values for state variables and retinol kinetic parameters for 50 theoretical children[1]

ID	BW (kg)	$[ROH]_p$ (µmol/L)	Plasma volume[2] (L)	M(5)[3] (µmol)	M(6) (µmol)	M(7) (µmol)	R(10,6) (µmol/d)	U(1) (µmol/d)	Liver VA[4] (µmol/g)
1	11.8	0.926	0.585	0.542	177		1.82	2.60	0.402
2	13.6	0.927	0.675	0.626	200		2.25	2.89	0.393
3	15.1	1.19	0.752	0.897	268		2.49	3.12	0.472
4	15.4	1.33	0.764	1.01	360		2.28	2.56	0.624
5	15.4	1.34	0.767	1.03	360		2.28	2.66	0.623
6	10.6	0.883	0.528	0.467	163		1.92	2.70	0.408
7	12.8	1.01	0.636	0.641	236		2.71	3.13	0.492
8	13.6	1.09	0.677	0.735	245		2.59	3.46	0.480
9	14.0	1.26	0.694	0.877	308		2.46	2.92	0.588
10	11.9	0.927	0.591	0.547	199		2.02	2.58	0.447
11	15.1	1.18	0.750	0.884	267		2.49	3.44	0.472
12	14.8	1.28	0.736	0.943	340	24.3	2.45	3.19	0.612
13	12.8	0.995	0.634	0.631	227	18.6	2.66	3.80	0.475
14	14.6	1.16	0.724	0.841	255	11.4	2.61	3.08	0.467
15	9.59	0.861	0.477	0.410	143	13.2	1.83	2.59	0.398
16	8.97	0.77	0.446	0.345	92	10.3	1.94	2.73	0.275
17	14.0	1.27	0.695	0.884	309	11.3	2.35	2.65	0.589
18	11.5	0.947	0.571	0.541	207	16.3	2.53	3.30	0.481
19	12.5	0.979	0.620	0.606	223	16.4	2.49	3.27	0.478
20	15.4	1.22	0.764	0.936	275	22.0	2.50	2.92	0.478
21	17.3	1.37	0.859	1.18	368	14.1	2.06	2.48	0.568
22	11.2	0.911	0.556	0.506	168	5.24	1.85	2.39	0.400
23	15.9	1.26	0.788	0.991	284	23.4	2.54	3.26	0.478
24	14.8	1.16	0.735	0.855	261	10.1	2.59	3.62	0.472
25	13.3	1.54	0.663	1.02	865		3.44	3.83	1.73
26	13.2	1.46	0.657	0.959	667		2.77	3.39	1.35
27	19.3	2.13	0.957	2.04	1772		12.3	16.8	2.45
28	21.9	2.36	1.09	2.57	1877		10.0	13.1	2.29
29	12.6	1.52	0.628	0.954	822		3.86	4.88	1.74
30	16.8	1.86	0.834	1.55	1588		4.17	5.46	2.52
31	18.9	2.10	0.940	1.98	1728		6.17	7.76	2.44
32	16.6	1.76	0.825	1.45	1203		3.06	3.82	1.93
33	17.5	1.81	0.871	1.57	1456		3.53	4.43	2.21
34	12.8	1.41	0.635	0.895	531		2.77	3.80	1.11
35	14.6	1.73	0.728	1.26	1079		4.43	5.57	1.97
36	17.1	1.87	0.848	1.59	1596		3.71	4.44	2.49
37	18.8	1.94	0.932	1.81	1638	28.4	4.85	5.96	2.33
38	22.2	2.37	1.10	2.62	1904	30.6	5.83	6.93	2.28
39	12.2	1.50	0.605	0.905	777	23.1	3.65	4.55	1.70
40	13.8	1.62	0.684	1.11	893	30.6	2.86	3.64	1.73
41	18.8	2.09	0.933	1.95	1728	38.6	12.1	14.1	2.45
42	10.1	1.35	0.502	0.676	406	23.1	2.41	2.79	1.07
43	16.7	1.83	0.830	1.52	1553	24.8	8.44	10.9	2.48
44	20.9	2.20	1.04	2.28	1838	28.8	11.2	13.2	2.35
45	18.6	1.94	0.923	1.79	1632	22.8	3.54	4.79	2.34
46	17.0	1.86	0.844	1.57	1590	21.2	7.16	9.70	2.50
47	15.9	1.74	0.788	1.37	1140	20.8	3.15	4.14	1.92
48	19.6	2.05	0.972	1.99	1680	28.9	3.38	3.89	2.29
49	13.1	1.45	0.652	0.945	570	28.4	1.88	2.63	1.16
50	12.2	1.39	0.608	0.845	475	19.7	1.92	2.34	1.04
GM	14.6	1.40	0.727	1.02	538	19.3	3.21	4.07	0.982
AM	14.9	1.46	0.742	1.14	779	21.0	3.76	4.76	1.27
Min	8.97	0.774	0.446	0.345	92	5.24	1.82	2.34	0.275
Max	22.2	2.37	1.10	2.62	1904	38.6	12.3	16.8	2.52

Supplemental Table 1 (continued)

ID	L(2,1)	DT(3)	L(4,3)	L(5,4)	L(6,5)	L(5,6)	L(10,6)	L(7,5)	L(5,7)
1	10.1	0.201	0.701	4.04	34.7	0.0958	0.0103		
2	12.6	0.205	0.781	4.75	35.5	0.0997	0.0113		
3	17.3	0.193	0.797	4.54	36.0	0.112	0.00929		
4	14.9	0.187	0.891	2.63	35.6	0.0937	0.00634		
5	14.0	0.206	0.859	3.71	24.2	0.0626	0.00633		
6	19.4	0.162	0.710	1.22	35.6	0.0903	0.0118		
7	13.3	0.189	0.866	2.78	24.7	0.0557	0.0115		
8	18.5	0.185	0.749	1.66	33.3	0.0894	0.0106		
9	13.5	0.155	0.844	2.34	23.5	0.0590	0.00799		
10	19.9	0.186	0.783	2.89	36.1	0.0892	0.0102		
11	17.9	0.160	0.723	2.44	39.6	0.122	0.00933		
12	12.8	0.194	0.768	14.9	34.9	0.0896	0.00721	30.4	1.18
13	19.3	0.177	0.701	10.8	25.9	0.0602	0.0117	58.7	1.99
14	16.3	0.191	0.845	12.1	24.5	0.0705	0.0102	39.3	2.91
15	15.7	0.163	0.708	12.0	29.1	0.0705	0.0128	60.9	1.90
16	16.8	0.213	0.712	9.26	20.1	0.0539	0.0210	55.1	1.84
17	12.9	0.221	0.887	14.6	37.2	0.0986	0.00759	15.6	1.22
18	17.9	0.207	0.764	6.51	29.9	0.0659	0.0122	41.1	1.36
19	19.2	0.219	0.762	12.8	22.1	0.0487	0.0112	25.0	0.927
20	19.3	0.229	0.855	10.1	37.1	0.117	0.00907	46.5	1.98
21	19.8	0.189	0.830	5.03	26.7	0.0797	0.00559	21.9	1.83
22	17.6	0.200	0.775	11.6	34.2	0.0920	0.0110	31.7	3.07
23	19.3	0.214	0.777	13.5	25.4	0.0797	0.00894	14.8	0.627
24	17.7	0.211	0.714	11.4	28.3	0.0825	0.00989	20.1	1.69
25	16.6	0.170	0.898	1.59	37.4	0.0401	0.00397		
26	12.8	0.213	0.817	4.26	27.9	0.0360	0.00416		
27	11.6	0.208	0.731	3.04	25.5	0.0225	0.00693		
28	19.1	0.191	0.765	3.28	24.2	0.0278	0.00533		
29	11.2	0.215	0.790	3.24	39.9	0.0417	0.00469		
30	17.1	0.165	0.765	1.96	29.3	0.0260	0.00263		
31	12.7	0.174	0.795	2.77	30.0	0.0307	0.00357		
32	18.5	0.202	0.801	4.60	31.9	0.0359	0.00255		
33	16.9	0.208	0.796	4.00	31.7	0.0318	0.00242		
34	19.0	0.178	0.730	2.46	26.7	0.0397	0.00522		
35	11.4	0.222	0.795	3.14	37.8	0.0401	0.00411		
36	16.6	0.227	0.835	4.15	23.6	0.0211	0.00232		
37	16.9	0.218	0.813	13.5	35.6	0.0363	0.00296	51.6	3.29
38	17.0	0.183	0.842	9.47	23.6	0.0294	0.00306	43.1	3.69
39	19.7	0.197	0.803	14.4	39.3	0.0411	0.00470	43.0	1.68
40	10.7	0.170	0.785	14.4	37.4	0.0433	0.00320	19.2	0.696
41	17.6	0.200	0.855	11.1	20.1	0.0156	0.00699	45.4	2.29
42	10.2	0.202	0.865	14.5	21.8	0.0304	0.00594	45.5	1.33
43	13.3	0.176	0.772	10.7	26.9	0.0208	0.00543	32.9	2.01
44	18.8	0.162	0.845	6.72	37.1	0.0400	0.00607	47.2	3.75
45	15.3	0.157	0.739	13.0	36.6	0.0379	0.00217	37.8	2.97
46	17.9	0.198	0.739	12.8	35.3	0.0304	0.00451	5.40	0.401
47	16.3	0.216	0.761	14.7	31.7	0.0354	0.00276	31.6	2.09
48	13.5	0.228	0.869	9.54	28.8	0.0322	0.00201	27.5	1.90
49	14.8	0.178	0.714	7.01	30.9	0.0479	0.00329	37.0	1.23
50	16.6	0.217	0.822	14.6	26.6	0.0432	0.00404	28.1	1.21
GM	15.7	0.194	0.789	6.02	30.1	0.0502	0.00597	31.9	1.67
AM	16.0	0.195	0.791	7.65	30.6	0.0571	0.00697	35.4	1.89
Min	10.1	0.155	0.701	1.22	20.1	0.0156	0.00201	5.40	0.401
Max	19.9	0.229	0.898	14.9	39.9	0.122	0.0210	60.9	3.75

[1]Values are data for state variables and model parameters for 50 theoretical children who had consumed an oral dose of labeled retinyl acetate, as well as data for geometric and arithmetic means and minimum and maximum values. State variables include body weight (BW), plasma retinol concentration {$[ROH]_p$}, plasma volume,

vitamin A mass in compartments 5, 6 and 7 [M(I), where M(6) represents vitamin A total body stores], intake of dietary preformed vitamin A [U(1)], vitamin A disposal rate [R(10,6)], and liver vitamin A concentration. Parameters (see Figure 2) are fractional transfer coefficients [L(I,J)s, or the fraction of retinol in compartment J transferred to compartment I each day], where L(3,2) = L(2,1) and L(0,3) = 1 – L(4,3), and delay time [DT(I), or the time spent in delay component I]. AM; arithmetic mean, GM; geometric mean, VA; vitamin A.
[2]Plasma volume (L) was estimated as body weight (kg) × 0.05 L/kg (Supplemental Reference 6).
[3]M(5) was calculated as plasma retinol concentration (µmol/L) × estimated plasma volume (L).
[4]Liver vitamin A was calculated as {[M(6) (µmol) × 0.8] ÷ [body weight (kg) × 0.03 ÷ 1000]} assuming that 80% of total body vitamin A is in the liver and that liver comprises 3% of body weight in children (28).

SUPPLEMENTAL TABLE 2 Known and model-predicted retinol state variables and kinetic parameters[1]

Parameter	Known value	Model-predicted value						
	Geometric mean (range)	Protocol 1	Protocol 2	Protocol 3				
				Scenario 1	Scenario 2	Scenario 3	Scenario 4	Scenario 5
State variables								
M(5), µmol	1.02 (0.345 – 2.62)	1.02	1.02	1.02	1.02	1.02	1.02	1.02
M(6), µmol	538 (92 – 1904)	535	537	445	534	489	573	505
M(7), µmol	19.3 (5.24 – 38.6)	10.4	10.0	6.93	19.5	10.3	13.6	10.2
U(I), µmol	4.07 (2.34 – 16.8)	5.11	4.90	8.63	5.09	8.67	4.08	6.85
R(5,4), µmol/d	3.21 (1.82 – 12.3)	4.07	3.91	6.90	4.07	6.93	3.26	5.48
R(6,5), µmol/d	30.6 (6.93 – 84.6)	34.2	32.4	35.8	44.2	32.3	30.5	32.1
R(5,6), µmol/d	27 (4.99 – 73.4)	30.1	28.5	28.9	40.1	25.4	27.3	26.6
R(10,6), µmol/d	3.21 (1.82 – 12.3)	4.09	3.92	6.90	4.07	6.93	3.26	5.48
R(7,5), µmol/d	32.2 (8.49 – 113)	14.1	9.15	6.11	14.6	9.47	5.60	5.64
R(5,7), µmol/d	32.2 (8.49 – 113)	13.9	9.08	6.11	14.6	9.47	5.60	5.64
Model parameters								
L(2,1), d^{-1}	15.7 (10.1 – 19.9)	22.6						
DT(3), d	0.194 (0.155 – 0.229)	0.204	0.255	0.250	0.258	0.252	0.264	0.249
L(4,3), d^{-1}	0.789 (0.701 – 0.898)	0.8	0.8	0.8	0.8	0.8	0.8	0.8
L(0,3), d^{-1}	0.201 (0.102 – 0.299)	0.2	0.2	0.2	0.2	0.2	0.2	0.2
L(5,4), d^{-1}	6.02 (1.22 – 14.9)	4.03	3.15	2.46	4.09	2.74	2.24	2.68
L(6,5), d^{-1}	30.1 (20.1 – 39.9)	33.6	31.8	35.2	43.5	31.7	30.0	31.5
L(5,6), d^{-1}	0.0502 (0.0156 – 0.122)	0.0562	0.0530	0.0650	0.0751	0.0518	0.0476	0.0527
L(10,6), d^{-1}	0.00597 (0.00201 – 0.210)	0.00764	0.00731	0.0155	0.00762	0.0142	0.00570	0.0109
L(7,5), d^{-1}	31.9 (5.40 – 60.9)	13.8	8.97	6.01	14.4	9.31	5.51	5.55
L(5,7), d^{-1}	1.67 (0.401 – 3.75)	1.34	0.908	0.882	0.750	0.923	0.410	0.554
Calculated parameters								
MST_{RBP}, d	0.522 (0.364 – 1.08)	0.541	0.572	0.656	0.502	0.617	0.710	0.622
T(5,5), d	0.318 (0.162 – 0.591)	0.250	0.259	0.147	0.25	0.147	0.312	0.186
T(6,5), d	168 (48 – 498)	131	137	64.4	131	70.5	175	92.1
T(7,5), d	6.06 (2.59 – 15.4)	2.55	2.56	1.00	4.79	1.48	4.18	1.86
T(SYS), d	172 (53.4 – 506)	134	140	65.5	136	72.1	179	94.1
t(5), d	0.0218 (0.0111 – 0.0426)	0.0211	0.0245	0.0243	0.0173	0.0244	0.0282	0.0270
v(5)	13.3 (3.28 – 36.8)	10.8	9.56	5.06	13.5	5.03	10.1	5.89
tt(5), d	12.8 (3.95 – 47.3)	12.3	14.6	12.9	10.1	14.3	17.8	15.9

[1]Data are geometric means (range) for the known values for state variables and kinetic parameters for 50 theoretical children as well as for model-predicted values for the datasets generated for 3 protocols. Protocol 1 included all 50 subjects at the extensive sampling times (n=36) and mean data were fit to the full model (Figure 2); protocol 2 included all subjects at the reduced number of sampling times (n=11); protocol 3 (scenarios 1 – 5) included all subjects at 4 d and 5 randomly-selected subjects at each of the remaining reduced sampling times. Data for protocols 2 and 3 were fit to the simplified model (Figure 2 inset). When fitting scenario 4 data, the geometric mean dietary intake was used to constrain the model as explained in Supplemental Methods. Shown are state variables, including compartment masses [M(I), µmol], dietary vitamin A intake [U(I), µmol/d; U(1) for the full model and U(3) for the simplified model], and transfer rates [R(I,J), or the mass (µmol) of retinol in compartment J transferred to compartment I each day]. Parameters are fractional transfer coefficients [L(I,J)s, or the fraction of retinol in compartment J transferred to compartment I each day] and delay time [DT(I), d; or the time spent in delay component I]. Other parameters calculated include the mean sojourn time to retinol binding protein [MST_{RBP}, d; or the sum of the turnover times for compartments 1, 2 and 4 plus delay time (d) in delay component 3, calculated as $L(2,1)^{-1} + L(3,2)^{-1} + DT(3) + L(5,4)^{-1}$, for the full model (Figure 2) or the delay time (d) in delay component 3 plus the turnover time in compartment 4, calculated as $DT(3) + L(5,4)^{-1}$, for the simplified model (Figure 2 inset)]. In addition, we calculated mean residence time [T(I,J), d; or the mean time tracer spends in compartment I after entering the system via compartment J], system residence time [T(SYS), d; or the total time tracer spends in plasma and in extravascular compartments 6 and 7 from the time it enters plasma until it is irreversibly lost from the system], transit time in plasma compartment 5 [t(5), d], recycling number [v(5), or the mean number of times a retinol molecule recycles to plasma from either compartments 6 or 7 before irreversibly being lost], and recycling time to plasma [tt(5), d]. Note that 3 significant figures were used for calculation of R(I,J)s, t(5), v(5) and tt(5) and this resulted in minor round-off differences. For additional information related to calculation of these kinetic parameters, see Cifelli et al. (6).

SUPPLEMENTAL TABLE 3 Vitamin A TBS predicted by population modeling and by RID, with composite RID equation coefficients and outcome evaluation, for theoretical children[1]

	Protocol 1	Protocol 2
Model-predicted TBS, µmol	535	537
RID-predicted TBS 4 d, µmol		
GM	522	541
range	80-2594	83-2690
Model-predicted $Fa \times S$ 4 d	0.81	0.84
Outcome evaluation (% of children)		
Within 25%	78	78
Within 50%	96	94
Within 75%	100	100

[1]Values are vitamin A TBS [M(6) in Figure 2] predicted by population modeling of the 2 datasets that included all 50 theoretical subjects at extensive (protocol 1) and reduced sampling times (protocol 2). Also shown are the geometric mean and range for TBS predicted by RID (Equation *2*; TBS = $Fa \times S \times 1/SA_p$) at 4 d and population model-predicted values for the composite RID coefficient $Fa \times S$ at 4 d, calculated as F(6) × {[F(5) ÷ M(5)] ÷ [F(6) ÷ M(6)]} where F(I) is tracer in compartment I at time *t* (4 d) and M(I) is vitamin A mass in compartment I. Compare these values for TBS with the geometric mean of the assigned values for all 50 theoretical children (538 µmol; range, 92 – 1904). Outcome criteria are percent of children whose 4 d predicted TBS was within 25, 50, or 75% of the assigned value. We specified that predictions were considered adequate when TBS 4 d was within 25% of the known value for ≥75% of children. GM, geometric mean; RID, retinol isotope dilution; TBS, total body stores.

Supplemental Figure 1

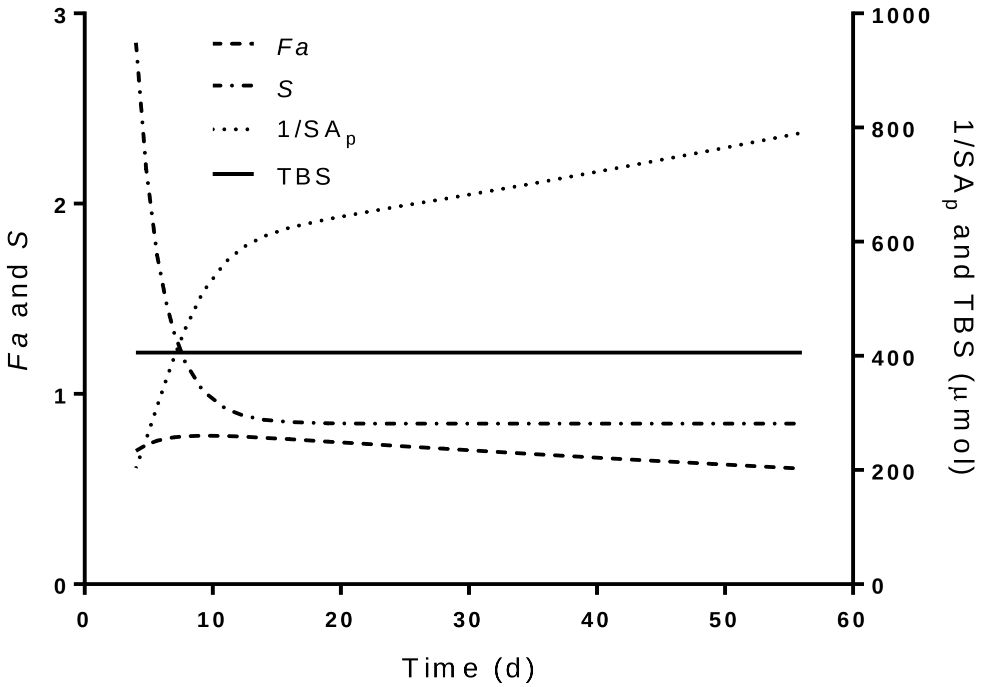

SUPPLEMENTAL FIGURE 1 Model-simulated values for *Fa, S* and $1/SA_p$ along with predicted values for TBS. When simulated values for *Fa, S* and $1/SA_p$ were used in the retinol isotope dilution equation (Equation *2;* TBS = *Fa* × *S* × $1/SA_p$) at any given time from 6 h to 56 d, they predicted time-invariant values for TBS. Shown are values for subject 42 (Supplemental Table 1). *Fa*, fraction of oral dose absorbed and retained in body stores at time *t*; *S*, the ratio of specific activity of retinol in plasma to that in stores; SA_p, plasma retinol specific activity; TBS, total body stores.

Supplemental Figure 2

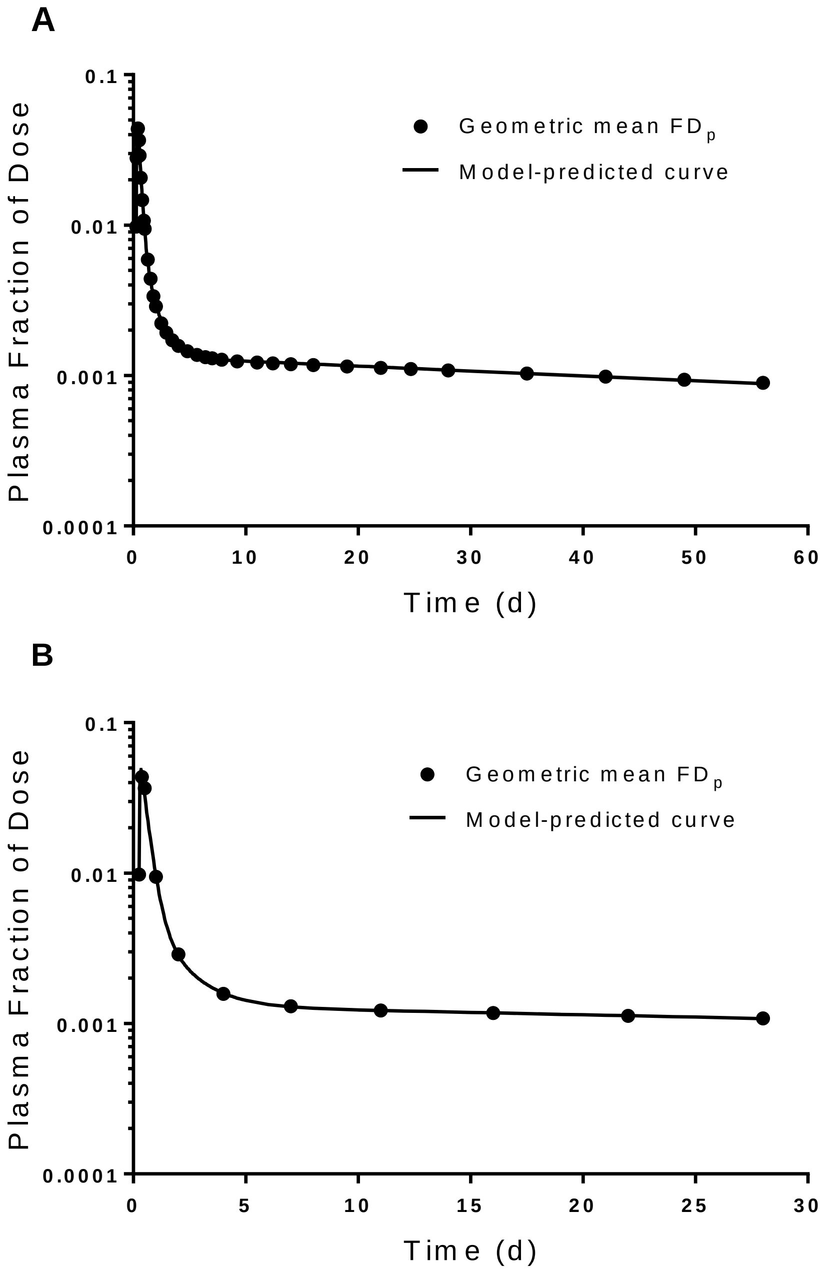

SUPPLEMENTAL FIGURE 2 Model-simulated composite plasma retinol response data versus time after ingestion of labeled retinyl acetate for 50 theoretical subjects generated using the extensive (panel A) and reduced sampling schedules (panel B). Panels A and B show geometric mean FD_p calculated using data for all 50 subjects at each time (n=36 for panel A and n=11 for panel B) and the model-calculated fit to the mean data. FD_p, fraction of dose in plasma.

Supplemental Figure 3

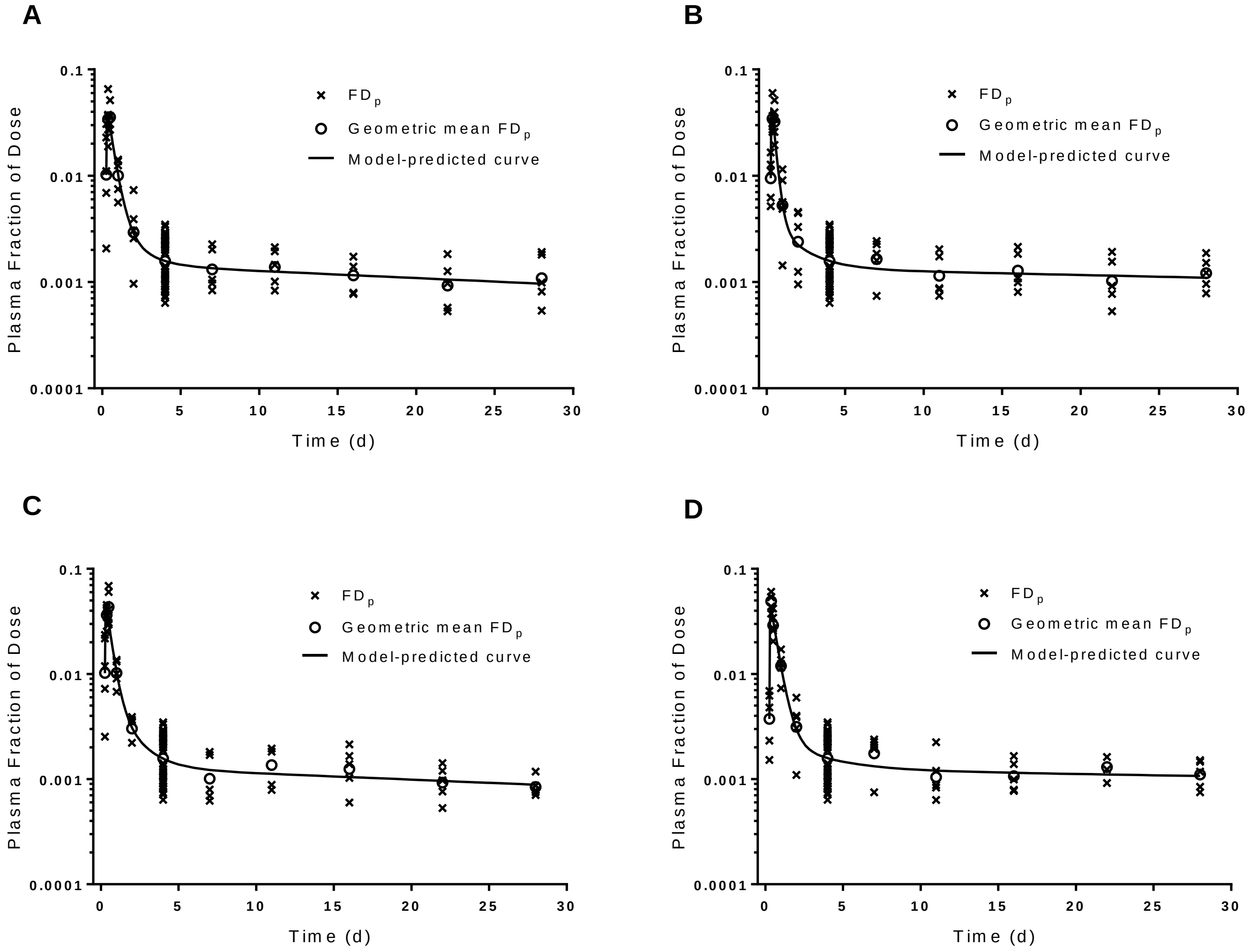

SUPPLEMENTAL FIGURE 3 Model-simulated composite plasma retinol tracer response data versus time for 4 of the 5 super-child datasets tested using protocol 3. Shown are “observed” FD_p for individual subjects (50 theoretical subjects at 4 d and 5 randomly-assigned children at each remaining time), the geometric mean FD_p at each time, and the model-calculated fit to the mean data. Protocol 3 / scenario 1 is shown in panel A, scenarios 2 – 4 are shown in panels B – D, respectively, and scenario 5 is shown in Figure 4. FD_p, fraction of dose in plasma.

Supplemental Figure 4

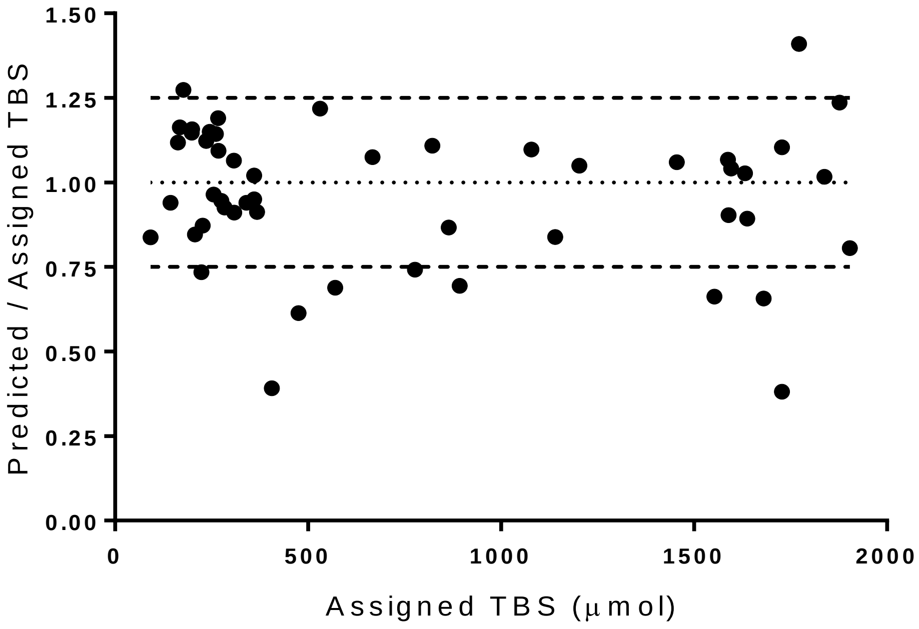

SUPPLEMENTAL FIGURE 4 Ratios of predicted to assigned values for vitamin A TBS versus assigned values for 50 theoretical children. Ratios were calculated for the super-child dataset (protocol 3 / scenario 5) which is shown in Figure 4; TBS was predicted at 4 d using the retinol isotope dilution equation (Equation *2*). Dashed lines indicate cutoffs for our evaluation criterion (ratios within 0.75 and 1.25 indicate 4 d predicted TBS was within 25% of the assigned value); a ratio of 1.0 is indicated by the dotted line. For this dataset, 4 d predictions were within 25% of the assigned value for 78% of subjects (Table 2). TBS, total body stores.

Supplemental Figure 5

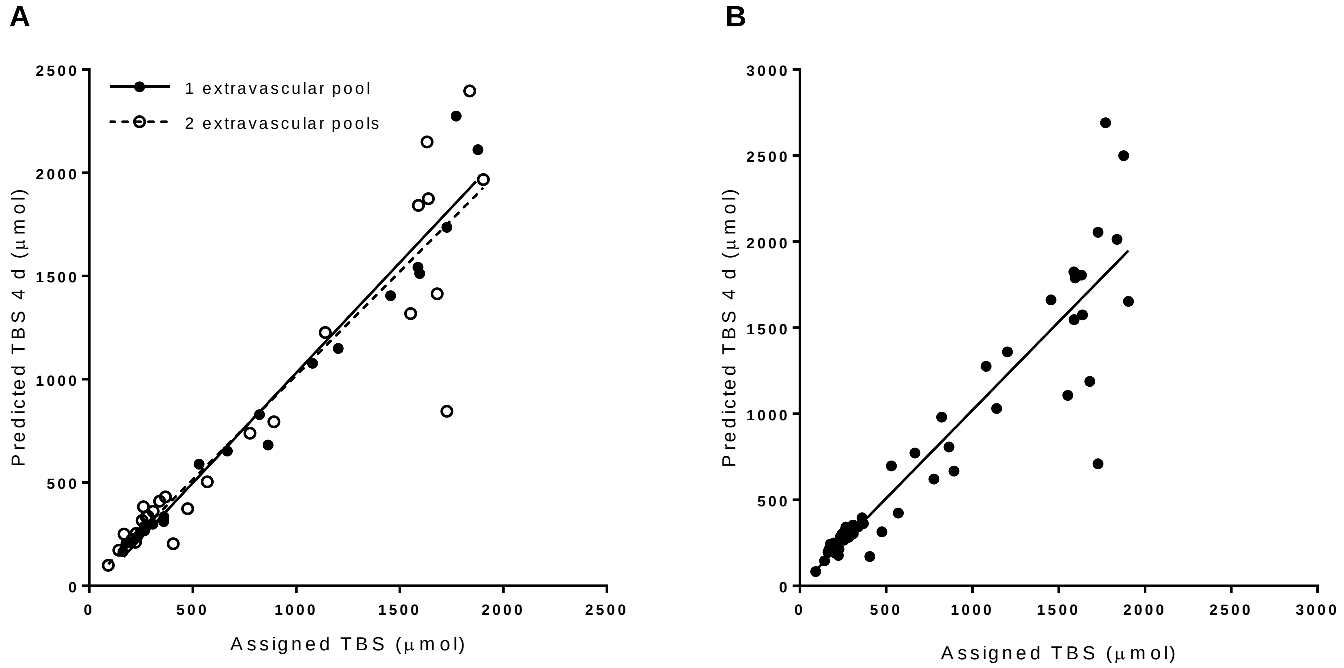

SUPPLEMENTAL FIGURE 5 Assigned values for vitamin A TBS for 50 theoretical subjects compared to TBS calculated at 4 d using the retinol isotope dilution equation (Equation *2*) for subjects with 1 (n=23) or 2 extravascular compartments (n=27) (panel A) as well as for all 50 subjects for protocol 2 (panel B). In panel A, TBS was calculated using the population value for the composite coefficient predicted at 4 d by the model with 1 or 2 extravascular compartment(s) that was fit to mean data for subjects with either 1 or 2 extravascular pool(s), respectively. In panel B, the composite coefficient was estimated for protocol 2's dataset where mean data for all 50 subjects were fit to a model with 2 extravascular compartments. Also shown are least squares regression lines: y = 1.1x – 35.9 (R^2 = 0.97; P < 0.0001) for subjects with 1 extravascular compartment and y = 1.0x + 11.7 (R^2 = 0.87; P < 0.0001) for subjects with 2 (panel A), and y = 1.0x – 4.4 (R^2 = 0.86; P < 0.0001) (panel B). TBS, total body stores.

Supplemental WinSAAM Deck

```
A SAAM31       SKM12  PROTOCOL 3 / SCENARIO 5 [14-MAY-2018]
CC SUPER-CHILD PROTOCOL
CC 50 CHILDREN AT 4 D, 5 CHILDREN/EACH REMAINING REDUCED SAMPLING TIME
CC SIMPLIFIED 5-COMPARTMENT MODEL FOR VITAMIN A KINETICS IN CHILDREN
CC PARAMETERS
H PAR
CC          VALUE          LOWER LIMIT    UPPER LIMIT
CC LABELED RETINYL ACETATE DOSE
CC IC(I)=INITIAL CONDITION (FRACTION OF DOSE) IN COMPARTMENT I AT TIME 0
   IC(3)=1.0
CC DT(I)=DELAY COMPONENT I (DAY)
CC DN(I)=NUMBER OF ELEMENTS IN DELAY COMPONENT I
   DT(3)     2.580157E-01  0.000000E+00   1.000000E+02
   DN(3)     8
CC L(I,J)=FRACTION OF J TRANSFERRED TO I PER DAY (DAY^-1)
CC ABSORPTION EFFICIENCY FIXED AT 80%
CC L(4,3)=FRACTIONAL ABSORPTION EFFICIENCY
   L(4,3)    0.8
CC L(0,3)=FRACTIONAL LOSS OF UNABSORBED TRACER
CC OUTPUT FROM DELAY COMPONENT EQUALS 1
   L(0,3)=1.0-L(4,3)
   L(5,4)    4.090305E+00  0.000000E+00   1.000000E+02
   L(7,5)    1.438871E+01  0.000000E+00   1.000000E+02
   L(5,7)    7.505053E-01  0.000000E+00   1.000000E+02
   L(6,5)    4.346064E+01  0.000000E+00   1.000000E+02
   L(5,6)    7.509678E-02  0.000000E+00   1.000000E+02
CC L(10,6)=TOTAL BODY STORES (TBS) FRACTIONAL CATABOLIC RATE
   L(10,6)   7.619576E-03  0.000000E+00   1.000000E+02
CC STEADY STATE SOLUTION
CC U(I)=VITAMIN A INPUT (UMOL/D)
CC M(I)=MASS OF VITAMIN A IN COMPARTMENT I (UMOL)
H STE
   U(3)     5.090070E+00  0              100
   M(5)     1.017134889
CC DATA
H DAT
C PLASMA RETINYL ACETATE-DERIVED RETINOL (COMPARTMENT 5)
CC         TIME (D)      GEOMETRIC MEAN  FRACTIONAL STANDARD DEVIATION
CC                       FRACTION OF DOSE
105                                      FSD=0.05
            0             0
            0.25          0.009428272
            0.375         0.034402374
            0.5           0.032217781
            1             0.005275378
            2             0.002396983
CC ALL SUBJECTS AT 4 D SO WEIGHT INCREASED
105                                      FSD=0.01
            4             0.001577891
105                                      FSD=0.05
            7             0.001643543
            11            0.001143295
```

	16	0.001279419
	22	0.00102381
	28	0.001209826

CC 115	TIME (D)	INDIVIDUAL SUBJECT FRACTION OF DOSE
	0.25	0.0110264
	0.25	0.0165724
	0.25	0.00622061
	0.25	0.012724
	0.25	0.00515092
	0.375	0.0599268
	0.375	0.0364237
	0.375	0.0313474
	0.375	0.0258659
	0.375	0.0272275
	0.5	0.0194295
	0.5	0.0394185
	0.5	0.0512674
	0.5	0.0258839
	0.5	0.0341543
	1	0.00488342
	1	0.0114617
	1	0.00142875
	1	0.00564657
	1	0.00904803
	2	0.000950885
	2	0.004463
	2	0.0045449
	2	0.00124483
	2	0.00329561
	4	0.0018719
	4	0.00210908
	4	0.00239075
	4	0.00230978
	4	0.00218076
	4	0.00200127
	4	0.00188514
	4	0.00203322
	4	0.00208544
	4	0.00186915
	4	0.0021714
	4	0.00230088
	4	0.00248508
	4	0.00266302
	4	0.00237636
	4	0.00347186
	4	0.00244892
	4	0.00240684
	4	0.00288017
	4	0.00280418
	4	0.00273098
	4	0.0020205
	4	0.0029408

4	0.00222977
4	0.00106308
4	0.00104442
4	0.00063741
4	0.000862348
4	0.000817314
4	0.000712516
4	0.000807704
4	0.000897741
4	0.000794617
4	0.0010793
4	0.000831292
4	0.000744214
4	0.000965669
4	0.00133082
4	0.00122413
4	0.0013978
4	0.00230432
4	0.00332345
4	0.00115187
4	0.000951812
4	0.000831266
4	0.000853262
4	0.0011197
4	0.00141053
4	0.00187769
4	0.00225941
7	0.00242576
7	0.0018506
7	0.000738999
7	0.00226198
7	0.00159813
11	0.00173605
11	0.000852381
11	0.000875441
11	0.00202969
11	0.000742914
16	0.000808351
16	0.00108268
16	0.000993643
16	0.00184282
16	0.0021392
22	0.000771638
22	0.000919032
22	0.000531382
22	0.00155645
22	0.00191783
28	0.000781948
28	0.00095957
28	0.0015164
28	0.00186894
28	0.00121885

Supplemental References

1. RStudio Team (2015). RStudio: Integrated Development for R. RStudio, Inc., Boston, MA URL http://www.rstudio.com/.
2. Olson JA. Serum levels of vitamin A and carotenoids as reflectors of nutritional status. J Natl Cancer Inst 1984;73:1439-44.
3. Palmer AC, West KP Jr, Dalmiya N, Schultink W. The use and interpretation of serum retinol distributions in evaluating the public health impact of vitamin A programmes. Public Health Nutr 2012;15:1201-15.
4. WHO Multicentre Growth Reference Study Group. WHO Child Growth Standards: Length/height-for-age, weight-for-age, weight-for-length, weight-for-height and body mass index-for-age: Methods and development. Geneva: World Health Organization, 2006.
5. Humphrey JH, Rice RL. Vitamin A supplementation of young infants. Lancet 2000;356:422-4.
6. Linderkamp O, Versmold HT, Riegel KP, Betke K. Estimation and prediction of blood volume in infants and children. Eur J Pediatr 1977;125:227–34.
7. Green MH, Green JB, Furr HC, Vitamin A Tracer Task Force. Analysis of stable isotope data to estimate vitamin A body stores. Vienna (Austria): International Atomic Energy Agency; 2008.
8. Tang G, Qin J, Dolnikowski GG, Russell RM. Short-term (intestinal) and long-term (postintestinal) conversion of β-carotene to retinol in adults as assessed by a stable-isotope reference method. Am J Clin Nutr 2003;78:259–66.
9. Thompson FE, Subar AF. Dietary assessment methodology. In: Coulston AM, Boushey CJ, Ferruzzi MG, editors. Nutrition in the prevention and treatment of disease. 3rd ed. Waltham (MA): Academic Press; 2013. p. 5-47.

10. Green MH and Green JB. Vitamin A intake and status influence retinol balance, utilization and dynamics in rats. J Nutr 1994;124:2477-85.
11. Haskell MJ, Jamil KM, Peerson JM, Wahed MA, Brown KH. The paired deuterated retinol dilution technique can be used to estimate the daily vitamin A intake required to maintain a targeted whole body vitamin A pool size in men. J Nutr 2011;141:428-32.
12. Ford JL, Green JB, Green MH. Should we restrict vitamin A intake, a minor contributor to plasma retinol turnover, when using retinol isotope dilution equations to estimate an individual's vitamin A status, or should vitamin A balance be maintained? J Nutr 2017;147:1483-6.
